# PbARID-associated chromatin remodeling events are essential for gametocyte development in *Plasmodium*

**DOI:** 10.1093/nar/gkae207

**Published:** 2024-03-30

**Authors:** Tsubasa Nishi, Izumi Kaneko, Shiroh Iwanaga, Masao Yuda

**Affiliations:** Department of Medicine, Mie University, Tsu 514-8507, Japan; Department of Medicine, Mie University, Tsu 514-8507, Japan; Research Institute for Microbial Diseases, Osaka University, Suita 565-0871, Japan; Department of Medicine, Mie University, Tsu 514-8507, Japan

## Abstract

Gametocyte development of the *Plasmodium* parasite is a key step for transmission of the parasite. Male and female gametocytes are produced from a subpopulation of asexual blood-stage parasites, but the mechanisms that regulate the differentiation of sexual stages are still under investigation. In this study, we investigated the role of PbARID, a putative subunit of a SWI/SNF chromatin remodeling complex, in transcriptional regulation during the gametocyte development of *P. berghei*. PbARID expression starts in early gametocytes before the manifestation of male and female-specific features, and disruption of its gene results in the complete loss of gametocytes with detectable male features and the production of abnormal female gametocytes. ChIP-seq analysis of PbARID showed that it forms a complex with gSNF2, an ATPase subunit of the SWI/SNF chromatin remodeling complex, associating with the male *cis*-regulatory element, TGTCT. Further ChIP-seq of PbARID in *gsnf2*-knockout parasites revealed an association of PbARID with another *cis*-regulatory element, TGCACA. RIME and DNA-binding assays suggested that HDP1 is the transcription factor that recruits PbARID to the TGCACA motif. Our results indicated that PbARID could function in two chromatin remodeling events and paly essential roles in both male and female gametocyte development.

## Graphical Abstract

**Graphical Abstract:**
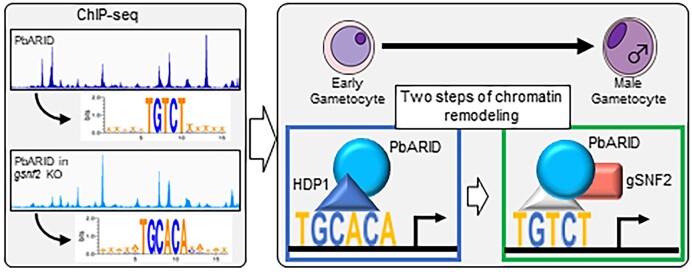

## Introduction

Malaria is one of the most serious infectious diseases in the world, which is caused by the infection of *Plasmodium* parasites (1). It typically causes fever, vomiting, and headaches, but in severe cases, it can also cause coma, jaundice, seizures, and even death (2). Various strategies, including vector control, chemotherapies, and vaccines, have been used over the last two decades to overcome malaria. However, the disease still occurs in over 200 million individuals and causes 500 000 deaths yearly (3).

*Plasmodium* parasites spread among people through mosquito bites. Thus, the transmission between vertebrate hosts and mosquitoes is an important process for malaria propagation. For the transmission from vertebrates to mosquitoes, the parasites go through sexual development (4,5). Sexual development begins with a subpopulation of the asexual blood-stage parasites differentiating into male and female gametocytes (6–8). After the gametocytes are ingested by mosquitoes through blood feeding, they egress from red blood cells to form microgamete and macrogamete in the mosquito midgut (9). The gametes then fertilize and develop into ookinetes, which invade the midgut epithelia, reach the basal lamina, and complete the transmission by forming oocysts (10,11). As the first step of *Plasmodium* sexual development, gametocytogenesis is essential for parasite transmission, and hence, elucidating the mechanism underlying gametocyte development is crucial for developing malaria control strategies such as transmission-blocking drugs and vaccines targeting the sexual stage parasites (12).

*Plasmodium* gametocyte development has been investigated in several studies based on transcriptomics and proteomics (13–19). In addition, recent studies on gametocyte-specific transcription factors significantly contribute to our knowledge of the mechanism underlying gametocyte development, mainly in *P. falciparum* and *P. berghei* (20). Gametocytogenesis is triggered by an AP2-family transcription factor, namely AP2-G (21,22). It is expressed in a subpopulation of blood-stage parasites, and disruption of its gene results in the complete loss of the ability of parasites to differentiate into gametocytes (23–25). Furthermore, the conversion of parasites into the sexual stage can be induced by the artificial activation of AP2-G (26,27). After gametocytogenesis is triggered, AP2-G2 induces genome-wide gene repression to support early gametocyte differentiation (28,29). Moreover, in *P. falciparum*, HDP1 and AP2-G5 were also reported to function during early gametocyte development (30,31).

The early gametocytes then differentiate into male and female gametocytes. For female gametocyte development, two transcriptional activators, AP2-FG and PFG, regulate the expression of female genes (32,33). In contrast, two transcriptional regulators, AP2-FG2 and AP2R-2, function together as a transcriptional repressor complex to support female development (34,35). In addition, transcriptional regulator genes for the zygote and ookinete development, such as *ap2-z* and *ap2-o*, are also transcribed in female gametocytes to prepare for post-fertilization development (36–39).

Compared with that during female gametocyte development, transcriptional regulation during male gametocyte development has been largely unexplored until some recent studies elucidated its mechanisms. A transcriptional regulator, gSNF2, was identified as a core subunit of the SWI/SNF chromatin remodeling complex, which participates in activating male genes by associating with the major male *cis*-regulatory element, TGTCT (40). Hence, the remodeling of the chromatin state is required for male gametocyte development. In addition to *gsnf2*, five male development regulator genes (*md1* through *md5*) were recently identified in *P. berghei* (41). Of these, *md1* was also identified as an essential factor for determining male fate in *P. falciparum* (42). Md1 is a cytoplasmic factor that interacts with ribonucleic granule proteins, and transcription of its gene is considered as a switch to control sex determination.

In our previous study, we reported that in *P. berghei*, one of the roles of AP2-G is to activate transcriptional regulator genes that are important for gametocyte development (43). These genes include most of the abovementioned transcriptional regulator genes. In this study, we investigate one of the transcriptional regulator genes in the AP2-G targets, *pbarid* (AT-rich interactive domain [ARID]-containing protein gene, PBANKA_0102400), during the gametocyte development of *P. berghei*. In a previous study, *pbarid* was identified as one of the regulator genes essential for male gametocyte differentiation (referred to as *md4*) (41). Moreover, the *P. falciparum* ortholog of PbARID was recently identified as a transcriptional regulator essential for microgametogenesis and macrogamete fertility (44). By investigating the role of PbARID, we demonstrated that PbARID forms a complex with gSNF2 and plays an essential role as a subunit of chromatin remodeling complexes in male differentiation and female development. Furthermore, we revealed that PbARID could function in two distinct chromatin remodeling events, and each event is associated with different DNA sequence motifs. Our results also suggested that the remodeling of the chromatin state is not only important for male gametocyte development but also for female gametocyte development.

## Materials and methods

### Ethical statement

All experiments in this study were performed according to the recommendations in the Guide for the Care and Use of Laboratory Animals of the National Institutes of Health to minimize animal suffering. All protocols were approved by the Animal Research Ethics Committee of Mie University (permit number 23–29).

### Parasite preparation

All parasites used in this study were inoculated in Balb/c or ddY mice. The *pbarid*(−), PbARID::mNG and PbARID::GFP parasites were derived from the WT ANKA strain, and all the other transgenic parasites were generated via the CRISPR/Cas9 system using Cas9-expressing parasites called PbCas9 (45). The growth rate of blood-stage parasites was assessed by counting infected red blood cells (RBCs) on Giemsa-stained smears every half-day after intraperitoneally injecting infected blood. Ookinete cultures and cross-fertilization assays were performed as previously described (36). Midgut oocysts were counted from the midgut of infected mosquitoes at 14 days post-infective blood meal.

### Generation of transgenic parasites

For tagging PbARID with fluorescent proteins, knockout of *pbarid*, and knockout of *gsnf2* for PbARID::GFP^C_^*^gsnf2^*^(−)^, the conventional homologous recombination method was used as previously reported (35,36,46). Briefly, for tagging experiments, two homologous regions of the *pbarid* locus were cloned into the *mNG*- or *gfp*-fusion vector to fuse *pbarid* in-frame with *mNG* or *gfp*. The vector was linearized using restriction enzymes before performing transfection experiments. For knockout experiments, targeting constructs, which contain a *hdhfr* expression cassette flanked with two homologous regions of a gene of interest, were prepared using overlap polymerase chain reaction (PCR). To generate the other transgenic parasites, a previously reported CRISPR/Cas9 system using PbCas9 parasites was used (45). Donor DNAs were constructed using overlap PCR, cloned into pBluescript KS(+), and amplified using PCR. Single guide RNA vectors were prepared by cloning target sequences using annealed oligos.

Transfection experiments were performed using Amaxa Basic Parasite Nucleofector Kit 2 (LONZA). All transfectants were selected by treating mice with 70 μg/mL pyrimethamine, which was added to their drinking water. Recombination was confirmed using PCR for tagging and knockout and using Sanger sequencing for mutations in promoters. Clonal parasites were obtained by limiting dilution. All primers used in this study are listed in Supplementary Table S6.

### Fluorescence-activated cell sorting (FACS) analysis

FACS analysis was performed using the LSR Fortessa (Becton Dickinson). Nuclei were stained with Hoechst 33342. The analyses were performed using peripheral blood from infected mice with a parasitemia of 2–3%. Cells were gated with forward scatter and Hoechst (450/50) fluorescence intensity. Gated cells were assessed for green fluorescent protein (GFP) (530/30) and red fluorescent protein (RFP) (582/15) fluorescence intensity.

### ChIP-seq and sequencing data analysis

The ChIP-seq experiments were performed as described previously (35). Briefly, parasites were enriched with gametocytes by adding sulfadiazine in the drinking water of infected mice. Whole blood was withdrawn from the infected mice and filtered using Plasmodipur. The blood samples were then fixed with 1% formalin at 30°C. After fixing, RBCs were lysed in ice-cold 1.5 M NH_4_Cl solution, and the residual cells were lysed in SDS lysis buffer. The lysates were sonicated using Bioruptor (Cosmo Bio) to shear chromatin and subjected to ChIP with anti‐GFP polyclonal antibodies (Abcam, ab290) immobilized on Dynabeads Protein A (Invitrogen). DNA fragments purified from the ChIP and input samples were used for library construction using KAPA HyperPrep Kit. The libraries were sequenced using Illumina NextSeq. Two biologically independent experiments were performed for each ChIP experiment.

The obtained sequence data were mapped onto the reference genome sequence of *P. berghei* ANKA, which was downloaded from PlasmoDB 55, using the Bowtie2 tool (47). Reads aligned at more than two different locations on the genome were removed from the mapping data. From the ChIP and input data, peaks were identified using the macs2 callpeak function with fold enrichment >3.0 and *q*-value <0.01 (48), and common peaks between the two experiments were used for further analysis. Binding motifs were predicted by analyzing the enrichment of motifs within 50 bp from peak summits using Fisher's exact test (37). Genes that possessed peaks within the 1200-bp upstream region from ATG were identified as target genes. Parameters for all programs were set to the default unless specified otherwise.

### RNA-seq and sequence data analysis

RNA extraction and RNA-seq experiments were performed as described previously (35). Briefly, gametocytes were enriched in the host as described above, and total RNA was extracted from the Plasmodipur-filtered whole blood of the infected mice using the Isogen II reagent (Nippon gene). From the total RNA, RNA-seq libraries were prepared using the KAPA mRNA HyperPrep Kit and sequenced using Illumina NextSeq. Three biologically independent experiments were performed for each sample. The obtained sequence data were mapped onto the reference genome sequence of *P. berghei* by HISAT2, setting the maximum intron length threshold to 1000 (49). The mapping data for each sample were analyzed using featureCounts (50) and compared using DESeq2 (51). The fragments per kilobase of transcript per million mapped reads (FPKM) for each gene were calculated from the featureCounts data, and genes with FPKM < 10 in all three datasets for WT were removed before the differential expression analysis. Genes in subtelomeric regions were also removed. For all programs, the parameters were set to the default unless specified otherwise.

### RIME and MS/MS data analysis

RIME was performed as previously described (35). Briefly, ChIP was performed as described for ChIP-seq analysis, and the IPed proteins were on-bead digested with 10 μl of trypsin (Promega) for overnight at 37°C. After the digestion, the beads were further incubated for 4 h at 37°C with additional 10 μl of trypsin added. The digested peptides were purified with C18 tip (GL-Science, Tokyo, Japan) and then subjected to nanocapillary reversed-phase LC-MS/MS analysis using a C18 column (12 cm × 75 μm, 1.9 μm, Nikkyo technos, Tokyo, Japan) on a nanoLC system (Bruker Daltoniks, Bremen, Germany) connected to a timsTOF Pro mass spectrometer (Bruker Daltoniks) and a modified nano-electrospray ion source (CaptiveSpray; Bruker Daltoniks).

The MS/MS data obtained by RIME was processed using DataAnalysis version 5.2 (Bruker Daltoniks), and proteins were identified using MASCOT version 2.7.0 (Matrix Science, London, UK) against the Uniprot_Plasmodium_berghei_ANKA_strain database (4948 sequences; 3412795 residues). Protease specificity was set for trypsin (C-term, KR; Restrict, P; Independent, no; Semispecific, no; two missed and/or nonspecific cleavages permitted). N-terminal Gln to pyro-Glu, and oxidation of methionine were considered as possible modifications. The mass tolerance for precursor ions and fragment ions were ±15 ppm and ±0.05 Da, respectively. The threshold score/expectation value for accepting individual spectra was *P* <0.05. Quantitative value was determined using Scaffold5 version 5.1.2 (Proteome Software, Portland, OR, USA) (52), and fold enrichment was calculated using Microsoft Excel. Proteins that were unique or more than fivefold enriched with *P*-value < 0.05 by two-tailed Student's t-test in PbARID::GFP compared to WT were identified as an interaction partner of PbARID.

### DIP-seq and sequencing data analysis

DIP was performed as described previously (43). Briefly, the DNA fragment encoding the homeodomain of HDP1 was cloned into the expression vector pGEX-6P-1 (Cytiva). *Escherichia coli* strain DH5α transformed with this plasmid was cultured for 12 h at 37°C. Next, expression of the GST-fused protein was induced by adding isopropyl β-D-thiogalactopyranoside (final concentration of 200 nM) in the culture and incubating for 9 h at 25°C. Recombinant homeodomain fused with GST was purified using glutathione-sepharose 4B resin (Cytiva), and eluted with 10 mM glutathione solution. Subsequently, the GST-fused homeodomain was incubated with *P. berghei* ANKA genomic DNA fragments in binding/washing buffer (10 μM ZnSO4, 2 mM MgCl_2_, 2 mM Tris–HCl at pH 7.4, 100 mM KCl and 10% glycerol) for 30 min. The protein/DNA solution was further mixed with glutathione-sepharose resin and incubated for 30 min. After incubation, the resin was washed three times with binding/washing buffer, and bound protein–DNA complexes were eluted with 10 mM glutathione solution. A sequencing library was prepared from the DNA fragments and sequenced using Illumina NextSeq. Before their use for DIP, genomic DNA fragments were also sequenced as an input. Analysis of the sequence data was performed same as that for ChIP-seq.

### ChIP-qPCR analysis

ChIP-qPCR analysis was performed as described previously (35). Briefly, ChIP was conducted via the same procedure as that for ChIP-seq analysis. Quantification of chromatin-immunoprecipitated and input DNA was performed using real-time qPCR using the TB Green Fast qPCR Mix (Takara) and Thermal Cycler Dice Real Time System II (Takara). All primers used are listed in Supplementary Table S6.

## Results

### PbARID is a putative subunit of a chromatin remodeling complex conserved in Apicomplexa

**Figure 1.**
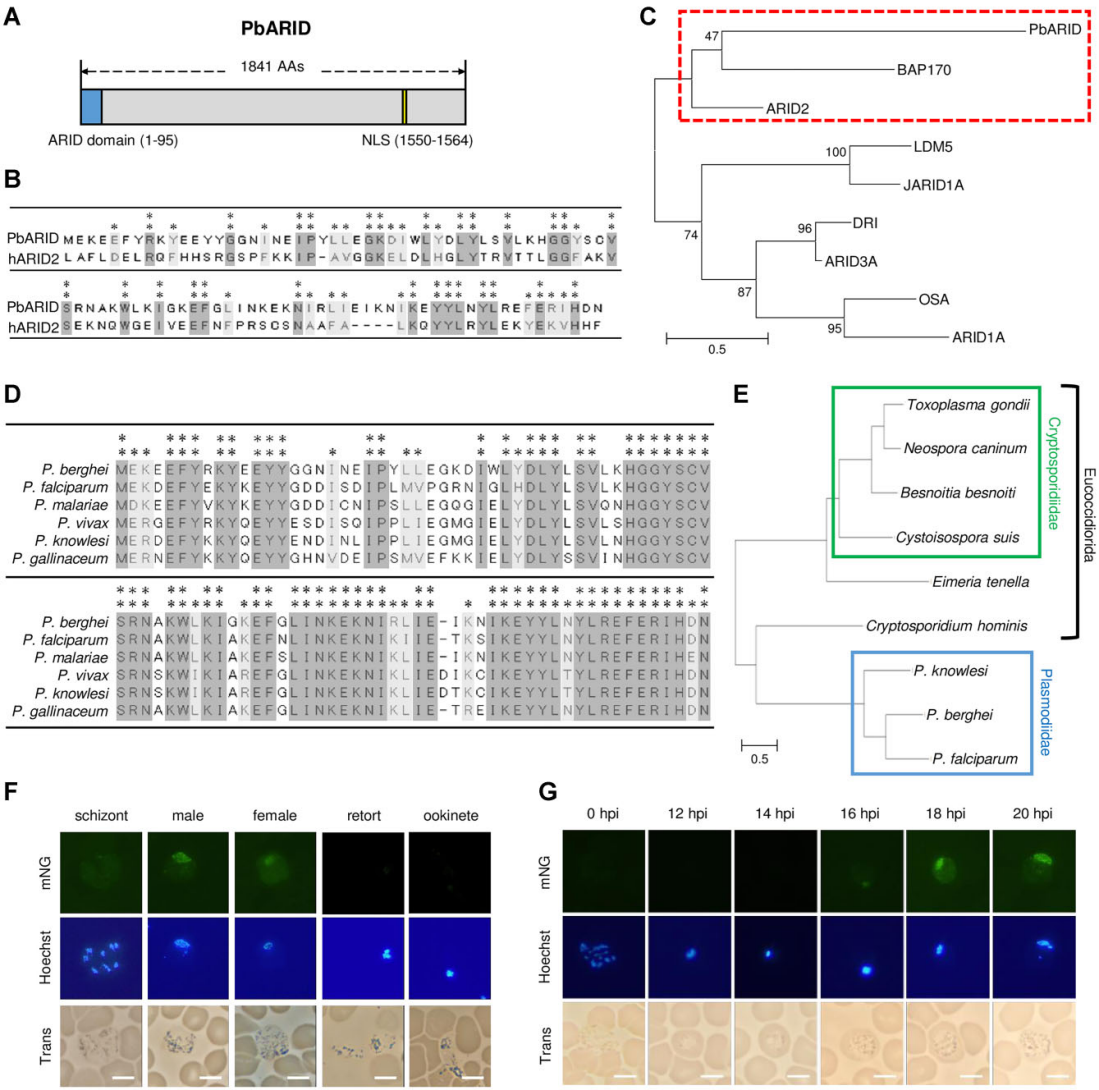
Features of PbARID and its expression during gametocyte development. (**A**) A schematic illustration of PbARID. The blue box shows the AT-rich interactive domain (ARID) domain. The nuclear localization signal (yellow box) was predicted using cNLS Mapper (http://nls-mapper.iab.keio.ac.jp/cgi-bin/NLS_Mapper_form.cgi). Amino acid positions for each feature are shown in brackets. (**B**) Alignment of amino acid sequences of PbARID and human ARID2 (hARID2). ** represents positions at which both sequences possess the same amino acid, whereas * represents positions with amino acid residues with similar properties. (**C**) A dendrogram of ARID domains from PbARID and ARID family proteins in human and *Drosophila* (human ARID [ARID1A, ARID2, ARID3A, and JARID1A] and *Drosophila* ARID [OSA, BAP170, DRI and LDM5]). Each ARID domain was determined using the simple modular architecture research tool. The amino acid sequences of ARID domains were aligned using the ClustalW program in Mega X. The tree was inferred from the alignment using the maximum likelihood method and Jones–Taylor–Thornton matrix-based model and was drawn to scale, with branch lengths measured according to the number of substitutions per site. The numbers above branches show the bootstrap values (1000 replicates). (**D**) Alignment of amino acid sequences of ARID domain from PbARID orthologs in *Plasmodium* species by the ClustalW program in Mega X (*Plasmodium berghei*, PBANKA_0102400; *Plasmodium falciparum*, PF3D7_0603600; *Plasmodium malariae*, PmUG01_11059200; *Plasmodium vivax*, PVP01_1145300; *Plasmodium knowlesi*, PKNH_1147100; and *Plasmodium gallinaceum*, PGAL8A_00132100). (**E**) Phylogenetic tree of PbARID and its putative orthologs in apicomplexan parasites (*P*. *berghei*, PbARID; *P*. *falciparum*, PF3D7_0603600; *P*. *knowlesi*, PKNH_1147100; *Cryptosporidium hominis*, XP_665701; *Eimeria tenella*, XP_013235762; *Toxoplasma gondii*, XP_018634911; *Neospora caninum*, XP_003885918; *Cystoisospora suis*, PHJ24466; and *Besnoitia besnoiti*, XP_029222326). The whole amino acid sequence for each protein was used for alignment. The tree was inferred as described in (C). The scale bar indicates a branch length measured according to the number of substitutions per site. (**F**) PbARID expression in asexual and sexual stages of the PbARID::mNG. Asexual-blood stages and gametocytes were assessed on peripheral blood smears. Retort-form and banana-shaped ookinetes were assessed in ookinete cultures at 16 and 20 h after starting the cultures, respectively. Nuclei were stained with Hoechst 33342. Scale bar = 5 μm. (**G**) Time-course fluorescent analysis of the PbARID::mNG during gametocyte development. Synchronized schizonts before injection were described as parasites at 0 h post-injection (hpi). Scale bar = 5 μm.

*pbarid* (PBANKA_0102400) is a target gene of AP2-G (43), and its transcription is upregulated via conditional induction of AP2-G in *P. falciparum* and *P. berghei* (26,27). *pbarid* encodes a protein that contains an ARID at its N-terminus and a monopartite nuclear localization signal near its C-terminus (Figure 1A). The ARID is a helix–turn–helix motif-based DNA binding domain conserved in a wide variety of eukaryotic species from fungi to vertebrates (53,54). In mammals, proteins with this domain are classified into seven subfamilies, namely ARID1 through ARID5, JARID1 and JARID2 (55). Of these, only ARID3 and ARID5 preferentially interact with AT-rich sequences, and the others have no clear sequence preference (56). These ARID proteins participate in the regulation of cell growth, differentiation, and development. To investigate a subfamily into which PbARID can be classified, we performed a protein–protein BLAST (blastp) search within ‘homo sapiens’ using the ARID domain (95 residues from the N-terminus) of PbARID as a query. The blastp result showed that the ARID domain of PbARID matched best with human ARID2 with an *E*-value of 7.0 × 10^−6^ (Figure 1B). Next, we constructed a dendrogram of ARID domains from PbARID and ARID family proteins in humans and *Drosophila*, the two species in which ARID proteins have been extensively characterized. The tree showed that PbARID is grouped into a clade with ARID2 and BAP170 (an orthologue of ARID2 in Drosophila), separate from the other ARID proteins (Figure 1C). ARID2 is a subunit of the chromatin remodeling complex, polycomb-associated BRG1/BRM-associated factor (PBAF), which is a type of SWI/SNF complex (57,58). These results suggested that PbARID could be related to the ARID2 subfamily.

The ARID domain of PbARID is highly conserved in *Plasmodium* species (Figure 1D). To evaluate whether PbARID is conserved in other Apicomplexan parasites, we performed the blastp search using the ARID domain of PbARID. The search identified proteins with an ARID domain from apicomplexan parasites such as *Toxoplasma* and *Cryptosporidium* (Supplementary Figure S1). Notably, the amino acids conserved among PbARID and the blastp-detected proteins contained most of the consensus amino acids for ARID domains. The phylogenetic tree of PbARID and the blastp-detected proteins was topologically consistent with the species tree of Apicomplexa, except that *Cryptosporidium* is closer to the clades of Sarcocystidae and Eimeriidae than that of Plasmodiidae in the species tree (Figure 1E). This result further suggested that these blastp-detected proteins are an ortholog of PbARID.

### PbARID is specifically expressed from early to mature gametocyte development

To investigate the cell stages in which PbARID functions, we generated a parasite line that expresses PbARID fused with mNeon Green (PbARID::mNG, Supplementary Figure S2A). No fluorescent signal was detected in any asexual stage parasites when the fluorescent analysis was performed using PbARID::mNG (Figure 1F). In contrast, nuclear-localized signals were detected in both male and female gametocytes (Figure 1F). We further assessed the fluorescence in ookinete cultures and observed no fluorescent signal in ookinetes (Figure 1F).

To evaluate the expression pattern of PbARID in detail during gametocyte development, we performed fluorescent analyses in a time-course manner using cell cycle-synchronized parasites. We intravenously injected mice with mature schizonts, which were cultured *in vitro* and collected via density gradient centrifugation, and observed fluorescence of PbARID::mNG every 2 h. Parasites showed no fluorescent signal from 0 h post-injection (hpi), *i.e*. schizonts before injection, to 14 hpi (Figure 1G). At 16 hpi, at which no parasites with sex-specific features have yet been observed in the Giemsa-stained smears, a small population of parasites started to express nuclear-localized fluorescence, and the signal became stronger at 18 hpi (Figure 1G). This result indicated that the expression of PbARID starts in early gametocytes before differentiation into male or female gametocytes. The fluorescent signals then continued until the maturation of gametocytes. This expression pattern of PbARID is consistent with the fact that *pbarid* is a target gene of AP2-G, because our previous result showed that the expression of mNG-tagged AP2-G starts at 12–14 hpi in *P. berghei* (43).

### Disruption of *pbarid* results in the complete loss of male gametocyte production

**Figure 2.**
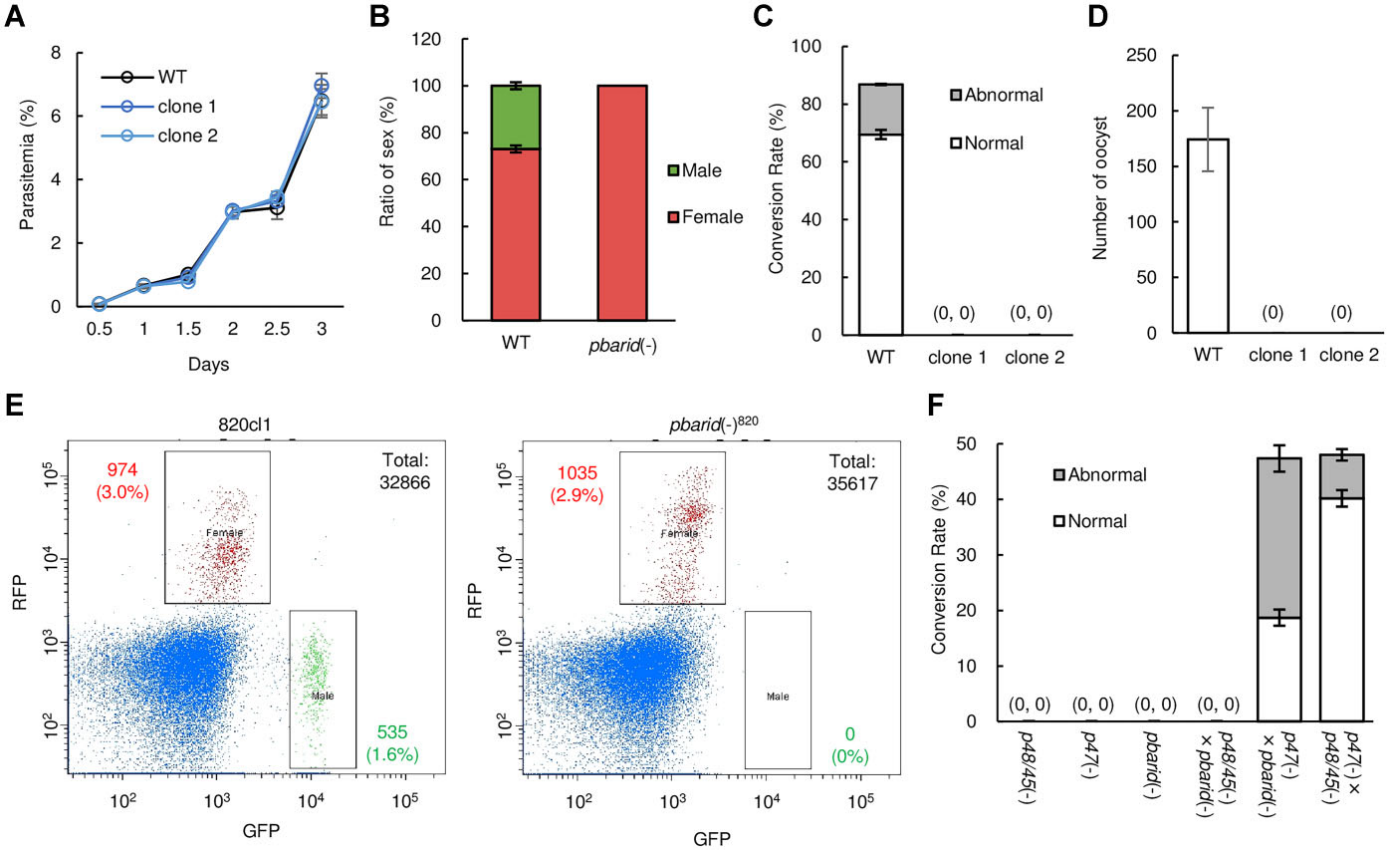
Knockout phenotype of *pbarid*. (**A**) Growth rate of blood-stage in wild-type (WT) and *pbarid*(−) parasites. Parasitemia was calculated by counting infected red blood cells on Giemsa-stained blood smears. The date for injection of infected blood into mice was set as day 0. (**B**) The ratio of male and female gametocytes in WT and *pbarid*(−) parasites. The number of gametocytes was assessed on Giemsa-stained blood smears in the gametocyte-enriched condition. Error bars indicate the standard error of the mean (*n* = 3). (**C**) The conversion rate of female gametocytes to normal ookinetes (banana-shaped; white box) and abnormal ookinetes (retort-form ookinetes and fertilized cells without apical protrusion; grey box). The number of female-derived cells was counted at 20 h after starting ookinete cultures. Error bars indicate the standard error of the mean (*n* = 3). (**D**) The number of midgut oocysts in WT and *pbarid*(−) parasites at 14 days post-infection. Error bars indicate the standard error of the mean (*n* = 20). (**E**) Fluorescence-activated cell sorting analysis of 820cl1 and *pbarid*(−)^820^ showing the numbers of female and male gametocytes as red and green fluorescent protein-positive parasites, respectively. Cells gated with forward-scatter and Hoechst are shown in the plot, and the number is indicated as ‘Total’ in the top right corner. Nuclei were stained with Hoechst 33342. (**F**) Cross-fertilization assay using *pbarid*(−), *p48/45*(−), and *p47*(−). The *p48/45*(−) and *p47*(−) parasites are male- and female-defective, respectively. The conversion rate for normal and abnormal ookinetes are shown in white and grey boxes, respectively. Error bars indicate the standard error of the mean (*n* = 3).

To investigate the role of PbARID in gametocyte development, we disrupted *pbarid* through a conventional homologous recombination method using a pyrimethamine resistance marker (*pbarid*[−], Supplementary Figure S2B). Two clonal lines were obtained from independent transfection experiments and used for phenotype analyses. The *pbarid*(−) parasites exhibited a growth rate that was comparable to that of the parental *Pb*ANKA strain (WT) (Figure 2A), indicating that PbARID plays no role in asexual blood-stage development. We next assessed the production of gametocytes by observing parasites in Giemsa-stained blood smears. In the assay, female gametocytes were observed, but no male gametocytes were observed in *pbarid*(−) (Figure 2B). Consistently, no exflagellation was detected in *pbarid*(−) after the activation of gametogenesis by exposing parasites to an ookinete culture medium (pH 8.2 and 20°C). As no male gametocyte was observed in the peripheral blood, *pbarid*(−) could not form any zygotes when cultured in an ookinete culture medium for both clones (Figure 2C). In addition, no oocysts were observed in the mosquito midgut 14 days after the blood meal (Figure 2D). To further confirm the absence of male gametocytes in *pbarid*(−), we knocked out *pbarid* in the gametocyte reporter line 820cl1 (*pbarid*[−]^820^), which have GFP and RFP expression cassettes under the control of male- and female-specific promoters, respectively. FACS analysis detected no GFP-positive parasites in the *pbarid*(−)^820^ parasites compared with the parental 820cl1 wherein approximately 3% of the parasites showed a GFP signal (Figure 2E). This confirmed that disruption of *pbarid* results in the complete loss of morphologically distinguishable male gametocytes. However, the single cell RNA-seq analysis of the *pbarid*-knockout parasite by Russell *et al.* demonstrated that the knockout parasite could produce immature male gametocytes at the transcriptomic level (41). Therefore, the results indicated that *pbarid*(−) is able to commit to male fate but does not undergo any further development to express recognizable male features. In *P. falciparum*, disruption of the *pbarid* ortholog only affected gametogenesis for male development (44). This difference could be due to the considerable difference in the gametocyte development for the two species; *i.e*. in *P. falciparum*, gametocytes take approximately 14 days to be fully matured and become crescent-shaped, developing an inner membrane complex (IMC).

We then performed cross-fertilization assays to evaluate whether female gametocytes of *pbarid*(−) can develop into ookinete when fertilized with normal male gametocytes. When *pbarid*(−) was crossed with parasites that produce infertile males (*p48/45*[−]) (59), no zygotes were observed, which was consistent with the absence of male gametocytes in *pbarid*(−) (Figure 2F). In contrast, when *pbarid*(−) was crossed with parasites that produce infertile females (*p47*[−]) (60), parasites could undergo fertilization at a ratio similar to that when *p48/45*(−) and *p47*(−) were crossed (Figure 2F). However, the ratio of conversion from female to banana-shaped ookinetes was only 19%, which was significantly smaller than the ratio for crossing *p48/45*(−) and *p47*(−), which was 40% (*P*-values = 4.9 × 10^−4^ using two-tailed Student's *t*-test) (Figure 2F). This result suggested that although female gametocytes of *pbarid*(−) showed normal morphology, they exhibit an abnormality that causes delay or arrest of ookinete development after fertilization. Collectively, these results indicated that PbARID plays a role in differentiation into male gametocytes and the development of female gametocytes. These results for the phenotype analyses of *pbarid*(−) are mostly consistent with a recent study by Russell *et al.*, except that the female gametocytes of the *pbarid*-knockout parasite did not show any defects based on their data (41). Meanwhile, in *P. falciparum*, disruption of the *pbarid* ortholog affects female gamete fertility, consistent with our result (44).

### PbARID is essential for regulating gametocyte transcriptome

**Figure 3.**
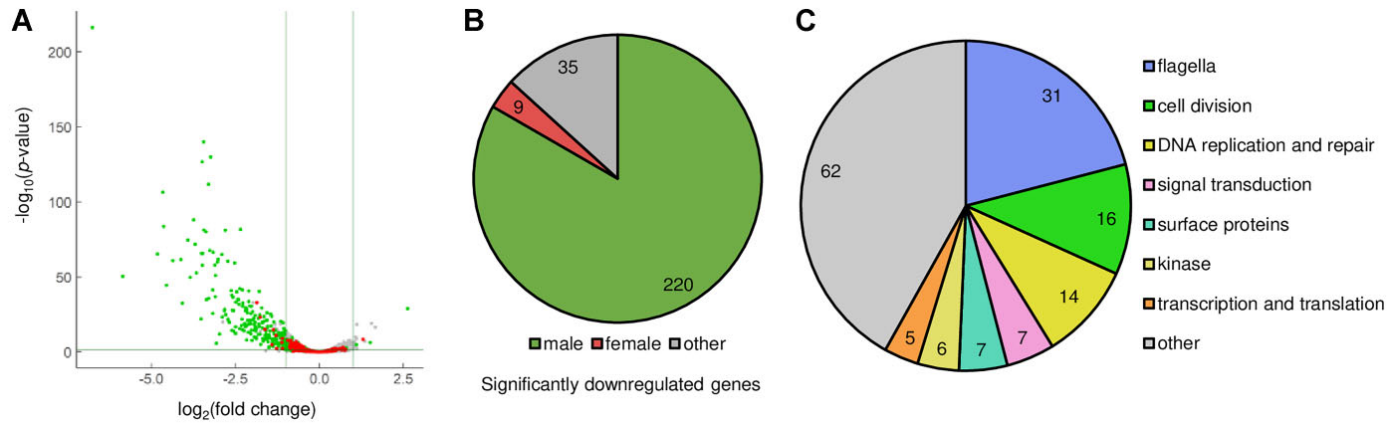
Differential expression analysis between wild-type (WT) and *pbarid*(−) gametocytes. (**A**) Volcano plot showing differential gene expression between WT and *pbarid*(−) gametocytes. Green and red dots represent male- and female-enriched genes, respectively. The horizontal line indicates a *P*-value of 0.05, and the two vertical lines indicate a log_2_(fold change) of 1 and − 1. (**B**) Classification of genes, which were significantly downregulated in *pbarid*(−), into male- and female-enriched genes and others. (**C**) Classification of the significantly downregulated genes that are functionally annotated on PlasmoDB into seven functional groups and ‘other.’

Because PbARID is nuclear-localized, we hypothesized that PbARID is involved in the transcriptional regulation of gametocytes. Accordingly, we performed a differential expression analysis between the gametocytes of WT and *pbarid*(−) to investigate its role in transcriptional regulation. Total RNA was harvested from gametocyte-enriched parasites by killing asexual blood-stage parasites using sulfadiazine treatment and used for RNA-seq analysis. Three independent samples were analyzed for each strain, and the sequence data were compared using DESeq2 (51). The analysis showed that in *pbarid*(−), 264 genes were significantly downregulated (log_2_[fold change] < −1, *P*-value adjusted for multiple testing with the Benjamini-Hochberg procedure [*P*-value adj] < 0.05), whereas only 15 genes were significantly upregulated (log_2_[fold change] > 1, *P*-value adj < 0.05) compared with those in WT (Figure 3A and Supplementary Table S1).

We then evaluated the effects of disrupting *pbarid* on male and female gametocyte transcriptome by comparing the significantly downregulated genes with the sex-enriched genes, which were previously identified using the sex-specific transcriptome data (19,35). Of the 264 significantly downregulated genes, 220 genes were male-enriched, which was consistent with the absence of male gametocytes in *pbarid*(−) (Figure 3B). They accounted for more than half of male-enriched genes (220/438) and included well-known male genes, such as *hap2*, *MiGS*, *p230* and *p230p* (60–64). In addition, many of the downregulated genes could be classified into the functional groups of major male features; in the classification of 148 downregulated genes that are functionally annotated on PlasmoDB (https://plasmodb.org/plasmo/app), the three largest groups were ‘flagella,’ ‘DNA replication and repair,’ and ‘cell division’ (Figure 3C). Along with the phenotype analysis, these results suggested that PbARID functions as a major factor for male gene activation.

### PbARID directly activates a majority of male genes

**Figure 4.**
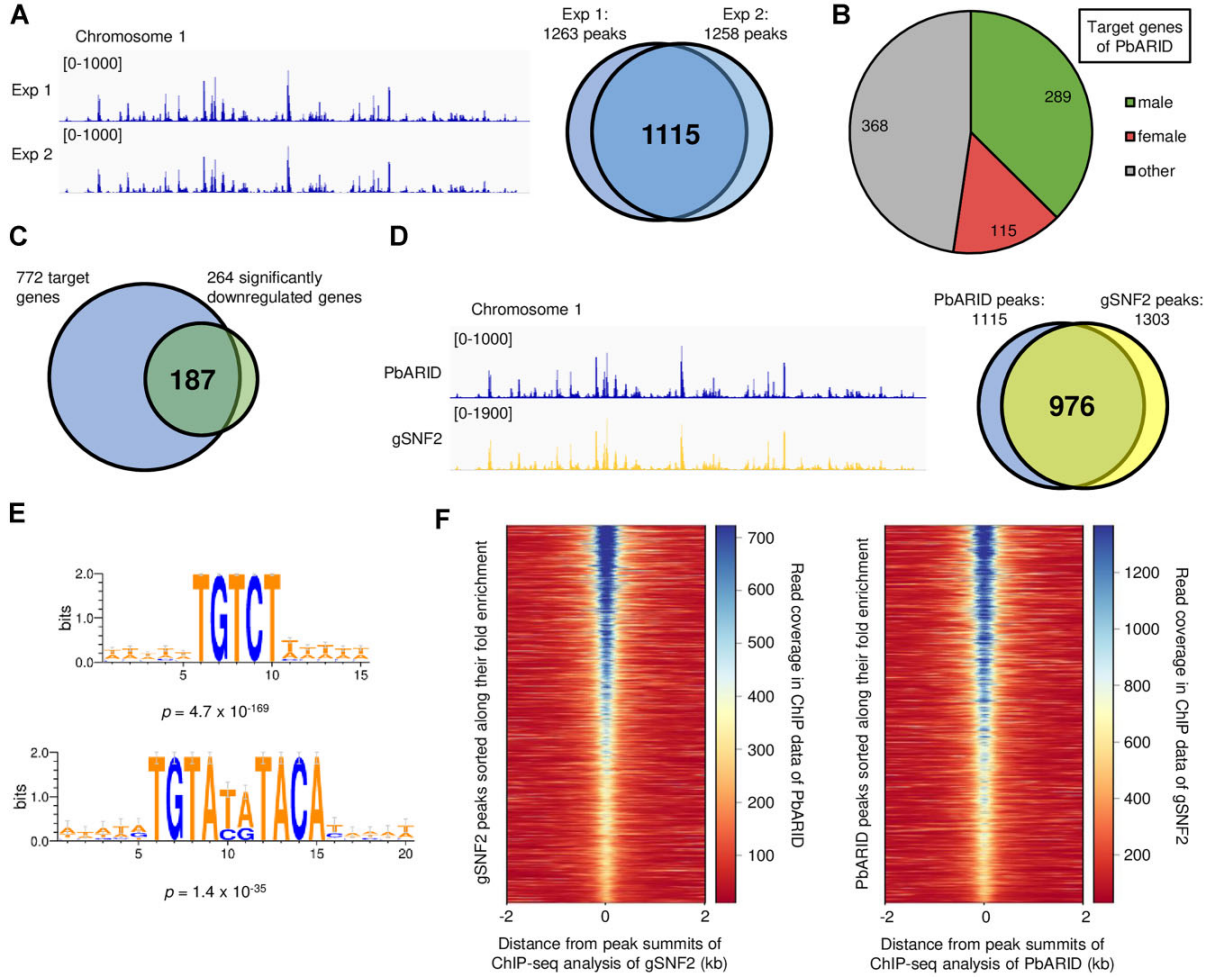
ChIP-seq analysis of PbARID. (**A**) Integrative Genomics Viewer (IGV) images from ChIP-seq Experiments 1 and 2 of PbARID on the whole chromosome 1. Histograms show row read coverage of the ChIP data at each base. The scales are shown in brackets. The Venn diagram on the right shows the number of peaks overlapped between Experiments 1 and 2. (**B**) Classification of PbARID target genes into sex-enriched gene sets. (**C**) Venn diagram showing overlap of the PbARID target genes and the significantly downregulated genes in *pbarid*(−). (**D**) IGV images showing ChIP-seq peaks of PbARID and gSNF2 on the whole of chromosome 1. The scales are shown in brackets. The Venn diagram on the right shows an overlap of peaks identified in ChIP-seq of PbARID and gSNF2. (**E**) Enrichment of TRTAYRTACA and TGTCT motifs within 50 bp from peak summits identified in the ChIP-seq of PbARID. The logos were depicted by WebLogo 3 (http://weblogo.threeplusone.com/). (**F**) Heatmaps showing coverage in ChIP-seq of PbARID at gSNF2 peaks (left) and that of gSNF2 at PbARID peaks (right). Peaks are aligned in ascending order of their fold enrichment values from the top of heat maps.

To investigate whether PbARID directly regulates the genes downregulated in *pbarid*(−), we performed chromatin immunoprecipitation followed by high-throughput sequencing (ChIP-seq) analysis using parasites that express GFP-fused PbARID (PbARID::GFP, Supplementary Figure S2C) and anti-GFP antibody. The IPed DNA fragments were sequenced via next-generation sequencing (NGS), and peaks were identified from the sequence data. Two independent experiments were performed, and 1263 and 1258 peaks were identified in Experiments 1 and 2, respectively (Supplementary Table S2A and S2B). Of these peaks, 1115 peaks overlapped between the two experiments, indicating high reproducibility of the ChIP-seq experiments (Figure 4A).

From the Chip-seq data, we searched for genes with a peak summit located within 1.2 kb upstream from the start codon and defined them as a target gene of PbARID. Through the search, we identified 772 target genes (Supplementary Table S2C). These target genes comprised the majority of male-enriched genes (289/444 male-enriched genes), indicating the role of PbARID in directly regulating male genes (Figure 4B). The targets also contained a considerable number of female-enriched genes, suggesting that PbARID plays a role in female development as well, which is consistent with the results of the phenotype analysis (Figure 4B). We then investigated whether these target genes were differentially expressed in *pbarid*(−) and revealed that the targets were enriched in the significantly downregulated genes (187/264 genes) with *P*-values of 2.5 × 10^−85^ using Fisher's exact test (Figure 4C). This result indicated that PbARID is involved in the transcriptional activation of its target genes.

Notably, the genome-wide peak pattern of the ChIP-seq of PbARID was almost identical to that of gSNF2, which we had previously reported (Figure 4D) (40). gSNF2 is an ATPase subunit of SWI/SNF chromatin remodeling complex expressed in gametocytes, and *gsnf2* knockout parasite (*gsnf2*[−]) showed impaired male gametocyte development (40). Of the peaks identified in the ChIP-seq of PbARID and gSNF2 (1115 and 1303 peaks, respectively), 976 peaks overlapped between them, which accounted for nearly 90% of the ChIP peaks of PbARID (Figure 4D). Consistently, we found the enrichment of the two motifs, TGTCT and TGTAYRTACA, which were gSNF2-associated motifs, within 50 bp of the peak summits in the PbARID ChIP-seq with *p*-values of 4.7 × 10^−169^ and 1.4 × 10^−35^ using Fisher's exact test, respectively (Figure 4E). Moreover, the intensity of each corresponding peak was highly consistent between the two ChIP-seq data; when a heatmap of read coverage for the ChIP data of PbARID was depicted, with location of the gSNF2 peak summits positioned at the center, higher read counts in PbARID ChIP-seq were observed at the gSNF2 peaks with higher fold enrichment (Figure 4F, left). The same was true for the ChIP-seq coverage of gSNF2 at the PbARID peaks (Figure 4F, right). Together with the results in the protein sequence analysis of ARID domains that indicated the relationship between PbARID and ARID2, it was strongly suggested that PbARID forms a complex with gSNF2 on the genome and together functions as the SWI/SNF chromatin remodeling complex.

### PbARID plays an additional role during gametocyte development independently from gSNF2

**Figure 5.**
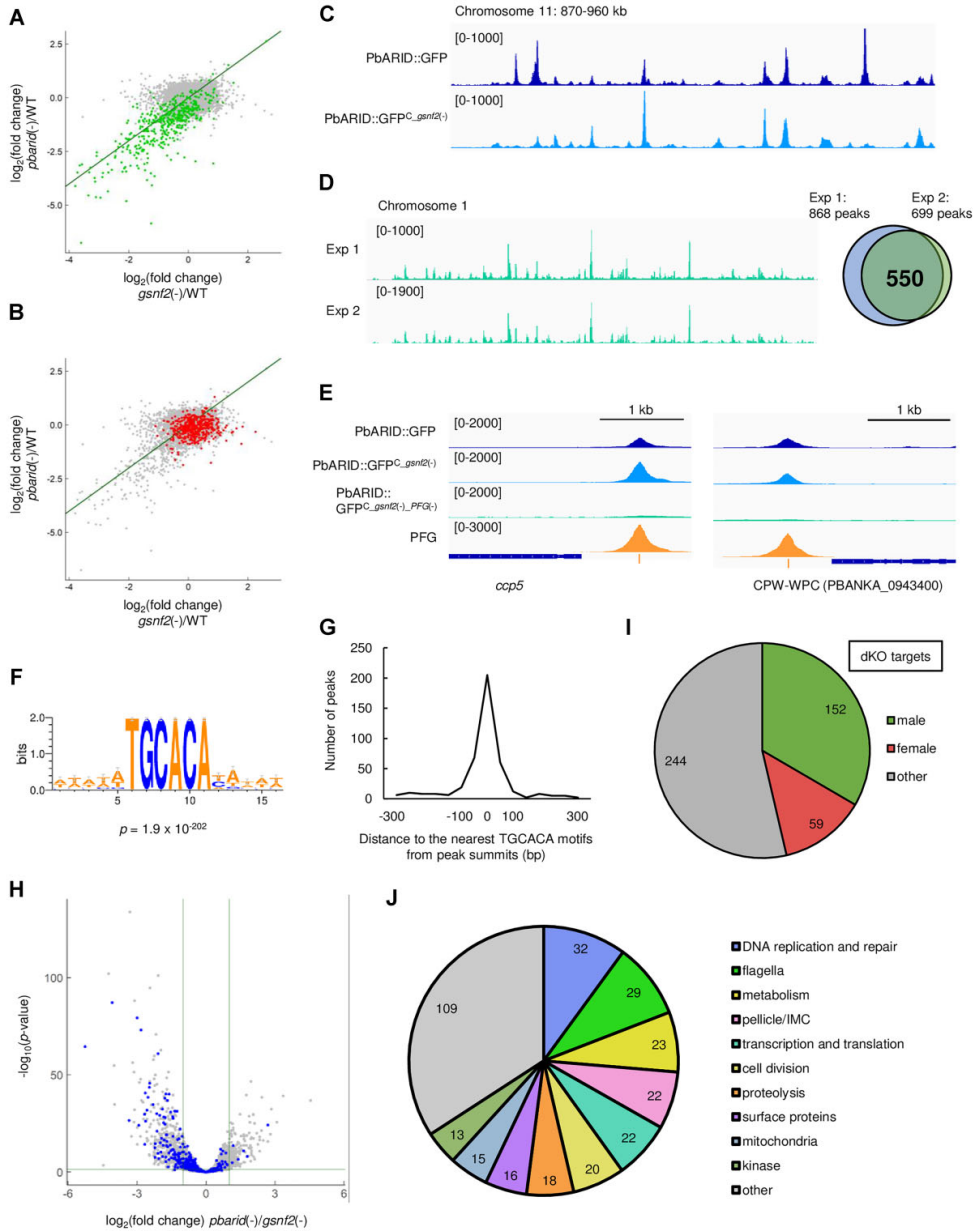
Function of PbARID in the absence of gSNF2. (**A**) The scatter plot showing the relationship between log_2_(fold change) for *pbarid*(−) versus wild-type (WT) and *gsnf2*(−) versus WT for each gene. Green dots represent male-enriched genes. The line shows a slope of 1 and an intercept of 0. (**B**) The scatter plot showing data mentioned in (A) for female-enriched genes, as indicated by red dots. (**C**) Integrative Genomics Viewer (IGV) images showing ChIP-seq peaks of PbARID in PbARID::GFP and PbARID::GFP^C_^*^gsnf2^*^(−)^ on a part of chromosome 11. Histograms show row read coverage of ChIP data at each base. The scales are shown in brackets. (**D**) IGV images from ChIP-seq Experiment 1 and 2 using PbARID::GFP^C_^*^gsnf2^*^(−)_^*^pfg^*^(−)^ on the whole chromosome 1. The scales are shown in brackets. The Venn diagram on the right shows the number of peaks overlapped between Experiments 1 and 2. (**E**) IGV images showing peaks that disappeared after *pfg* disruption. ChIP-seq peaks of PbARID in PbARID::GFP, PbARID::GFP^C_^*^gsnf2^*^(−)^, and PbARID::GFP^C_^*^gsnf2^*^(−)_^*^pfg^*^(−)^ and peaks of PFG are shown. Yellow bars indicate the positions of TGTAYRTACA motifs. The scales are shown in brackets. (**F**) A DNA motif enriched within 50 bp from peak summits identified in the ChIP-seq using PbARID::GFP^C_^*^gsnf2^*^(−)_^*^pfg^*^(−)^. The logo was depicted by WebLogo 3. (**G**) Distance between peak summits identified in the ChIP-seq using PbARID::GFP^C_^*^gsnf2^*^(−)_^*^pfg^*^(−)^ and the nearest TGCACA motifs. (**H**) Volcano plot showing differential gene expression between gametocytes of *pbarid*(−) and *gsnf2*(−). Blue dots represent the PbARID target genes in PbARID::GFP^C_^*^gsnf2^*^(−)_^*^pfg^*^(−)^. The horizontal line indicates a *P*-value of 0.05, and the two vertical lines indicate log_2_(fold change) of 1 and −1. (**I**) Classification of PbARID target genes in PbARID::GFP^C_^*^gsnf2^*^(−)_^*^pfg^*^(−)^ into sex-enriched gene sets. (**J**) Classification of functionally annotated target genes of PbARID in PbARID::GFP^C_^*^gsnf2^*^(−)_^*^pfg^*^(−)^ into ten functional groups and ‘other.’

The ChIP-seq analysis of PbARID indicated the cooperation of PbARID and gSNF2 in the transcriptional regulation of their target genes. However, their knockout phenotypes are not the same; *gsnf2*(−) produces immature male gametocytes, whereas *pbarid*(−) completely loses the ability to differentiate into male gametocytes. In addition, female gametocyte development was affected in *pbarid*(−) but not in *gsnf2*(−). Consistent with the differences in their phenotype, the results in the differential expression analyses for *pbarid*(−) versus WT and *gsnf2*(−) versus WT were also different; more than 70% of male- and female-enriched genes had lower fold change values in *pbarid*(−) versus WT than in *gsnf2*(−) versus WT (Figure 5A and B). Furthermore, a direct comparison of the RNA-seq data between *pbarid*(−) and *gsnf2*(−) identified 516 genes that were significantly downregulated in *pbarid*(−) compared with those in *gsnf2*(−), which contained 94 male- and 112 female-enriched genes (Supplementary Table S3). However, when compared with those in WT, the downregulation of female-enriched genes was barely detected in *pbarid*(−). This was probably because the significant difference in the male transcriptome between WT and *pbarid*(−) masked the less significant changes in the female transcriptome. Compared with those in *gsnf2*(−), the downregulation of female-enriched genes in *pbarid*(−) only became detectable due to less significant differences in their male transcriptomes.

From this result, we considered that PbARID plays an additional role in transcriptional regulation during gametocyte development. To explore the interaction of PbARID with factors other than gSNF2, we performed a ChIP-seq of PbARID in the *gsnf2*-knockout parasite. For this purpose, PbARID::GFP was generated with the CRISPR/Cas9 system using Pbcas9 (PbARID::GFP^C^, Supplementary Figure S3A), and then, *gsnf2* was disrupted in the PbARID::GFP^C^ (PbARID::GFP^C_^*^gsnf2^*^(−)^, Supplementary Figure S3B). The ChIP-seq analysis showed a different pattern of peaks compared with that in the ChIP-seq using PbARID::GFP (Figure 5C). Around these peak summits, the TGTCT motif was no longer enriched (*P*-value = 0.25). As previously reported, number of male and female gametocytes produced in the *gsnf2*-knockout parasites is comparable to that in WT (40). Hence, disappearance of the TGTCT motifs in the PbARID ChIP-seq was not derived from population changes, such as absence of male gametocytes. This result confirmed that PbARID is recruited by gSNF2 as a subunit of the SWI/SNF complex on the TGTCT motifs. In contrast, the enrichment of the TGTAYRTACA was still observed with a *P*-value of 8.7 × 10^−104^ using Fisher's exact test. In our previous study, we showed that the female-specific transcriptional activator, PFG, is responsible for recognizing the TGTAYRTACA motifs (33). Therefore, PbARID is recruited to the TGTAYRTACA motifs by PFG independent of gSNF2. In addition to the ten-base motif, the TGCACA motif was also enriched around the peak summits identified in the ChIP-seq using PbARID::GFP^C_^*^gsnf2^*^(−)^ (*P*-value = 3.0 × 10^−117^), suggesting that it could be another important PbARID-associated sequence motif.

To thoroughly investigate the property of PbARID binding regions other than those on the known motifs, we knocked out both *gsnf2* and *pfg* in PbARID::GFP^C^ using CRISPR/Cas9 (PbARID::GFP^C_^*^gsnf2^*^(−)_^*^pfg^*^(−)^, Supplementary Figure S3C). According to previous studies, *gsnf2*- and *pfg*-knockout would cause independent effects on gametocyte development in *P. berghei*; *i.e. gsnf2*-knockout affects male development but not female development whereas *pfg*-knockout shows abnormality in female development but not in male development. Consistently, the PbARID::GFP^C_^*^gsnf2^*^(−)_^*^pfg^*^(−)^ produced morphologically mature male and female gametocytes, but they were both infertile due to disruption of *gsnf2* and *pfg*, respectively. We performed two independent ChIP-seq analysis using PbARID::GFP^C_^*^gsnf2^*^(−)_^*^pfg^*^(−)^ and identified 868 and 699 peaks, of which 550 peaks overlapped between the two experiments (Figure 5D, Supplementary Table S4A, and S4B). As expected, peaks around the TGTAYRTACA motif mostly became undetectable after the disruption of *pfg*, and its enrichment in peak regions was significantly decreased (*p*-value = 7.5 × 10^−3^) (Figure 5E). However, the TGCACA motif was significantly enriched in the peak regions with a *P*-value of 1.9 × 10^−202^ using Fisher's exact test (Figure 5F). In addition, the TGCACA motif was found within 300 bp from the summit of 424 peaks (77% of the peaks), and most of them were located within 100 bp from the summit (Figure 5G). These results suggested that PbARID is indeed associated with the TGCACA motif. We further assessed genome-wide peak location and identified 455 target genes of PbARID in PbARID::GFP^C_^*^gsnf2^*^(−)_^*^pfg^*^(−)^ (dKO targets, Supplementary Table S4C). The dKO targets included 102 genes that were significantly downregulated in *pbarid*(−) compared with those in *gsnf2*(−), with a *P*-value of 2.0 × 10^−11^ using Fisher's exact test (Figure 5H). In contrast, the genes downregulated in *gsnf2*(−) compared with those in *pbarid*(−) only contained seven of the dKO targets. Therefore, we considered that as the additional role, PbARID activates genes downstream of the TGCACA motif, from which the phenotypic differences in the gametocyte development between *pbarid*(−) and *gsnf2*(−) could be derived.

To evaluate the role of PbARID on the TGCACA motif in gametocyte development, we classified the 319 functionally annotated dKO targets into 10 functional groups and ‘other.’ The groups for male-related functions, ‘DNA replication and repair’ and ‘flagella,’ were the two major groups that contained 32 and 29 genes, respectively (Figure 5J). Furthermore, another male-related functional group, ‘cell division,’ also contained a significant number of genes. Most of these male genes are also included in the targets of the TGTCT motifs. Therefore, this result suggested that some male genes are regulated by the following two different complexes containing PbARID: one with gSNF2 on the TGTCT motif and the other on the TGCACA motif, probably with an unknown remodeler. In addition to these male-related genes, the dKO targets also contained several genes in female-related groups, such as ‘pellicle/IMC’ and ‘mitochondria.’ Moreover, the targets included a considerable number of both sex-enriched genes, *i.e*. 152 male- and 59 female-enriched genes (Figure 5I), implying the role of the TGCACA motif in both male and female gametocyte development.

### The TGCACA motif functions as a *cis*-regulatory element for activating male genes

**Figure 6.**
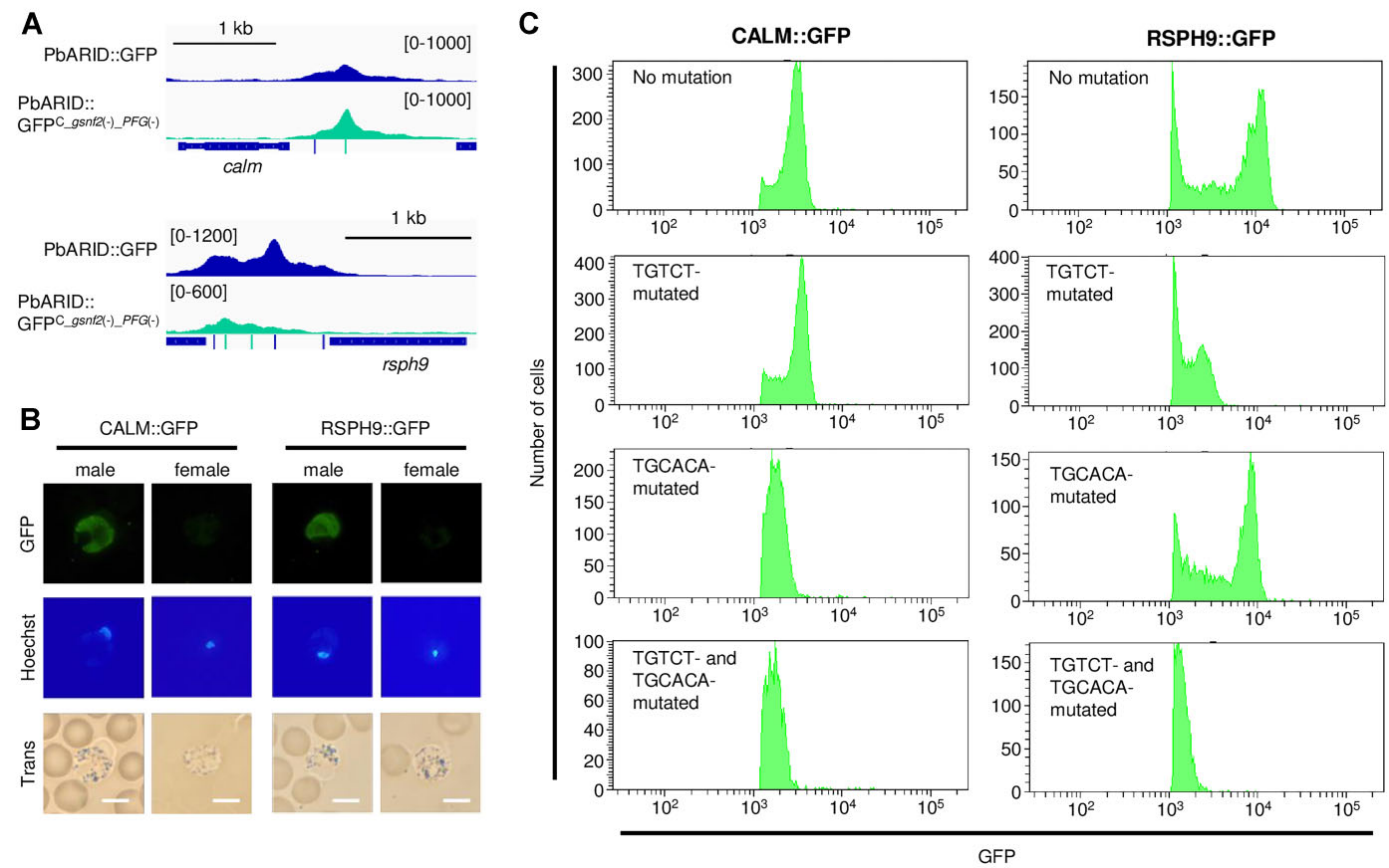
Role of the TGCACA motif as a *cis*-regulatory element for activating male genes. (**A**) ChIP-seq peaks of PbARID in PbARID::GFP and PbARID::GFP^C_^*^gsnf2^*^(−)_^*^pfg^*^(−)^ upstream of *calm* (top) and *rsph9* (bottom). Histograms show row read coverage of ChIP data at each base. Locations of TGTCT and TGCACA motifs are indicated as blue and green bars, respectively. The scales are shown in brackets. (**B**) Expression of CALM and RSPH9 in male and female gametocytes, observed using CALM::GFP and RSPH9::GFP parasites and fluorescence microscopy. Nuclei were stained with Hoechst 33342. Scale bar = 5 μm. (**C**) Fluorescence-activated cell sorting analysis of CALM::GFP (left) and RSPH9::GFP (right) before and after introducing mutations on the TGTCT and TGCACA motifs in their upstream peak regions. GFP-positive parasites were selected from cells gated with forward scatter and Hoechst. Nuclei were stained with Hoechst 33342.

To investigate the function of TGCACA motif as a *cis*-regulatory element and the relationship between the TGTCT and TGCACA motifs in male gametocyte development, we performed reporter assays using endogenous loci. As a target for the reporter assays, we selected the following two genes: *calm* (PBANKA_1421000, a putative calmodulin gene) as a gene significantly downregulated in *pbarid*(−) but only slightly in *gsnf2*(−) (log_2_[fold change] = −2.3 and −0.92, respectively) and *rsph9* (PBANKA_1431500, a gene encoding a putative radial spoke head protein 9 homolog, a component of radial spokes which control axonemal dynein activity) (65–67) as a gene downregulated in both *pbarid*(−) and *gsnf2*(−) (log_2_[fold change] = −6.8 and −3.6, respectively) (Supplementary Table S1). We first tagged each of these genes with *gfp* to assess their expression using FACS (CALM::GFP and RSPH9::GFP, Supplementary Figure S4A and S4B). Then, we mutated the TGTCT and TGCACA motifs, either or both, within the peak regions located upstream of *calm* and *rsph9* to assess the roles of these two *cis*-regulatory elements in the activation of male genes (Figure 6A, Supplementary Figure S4C and S4D).

The expression of the *gfp*-tagged genes was analyzed using FACS. Before introducing mutations, both CALM and RSPH9 were specifically expressed in the male gametocytes, which was consistent with the sex-specific transcriptome in which these genes are male-enriched (Figure 6B). For *calm*, disruption of the TGTCT motif did not show a distinct change in the expression of CALM (Figure 6C, right). However, mutations in the TGCACA motifs resulted in a significant reduction (approximately two-fold) of the GFP signal intensity, and mutations in both motifs showed a similar result (Figure 6C, right). Therefore, this indicates that the TGCACA motif functions as a major *cis*-regulatory element for the activation of *calm*. For *rsph9*, mutations in the TGTCT motifs resulted in a significant reduction (approximately five-fold) of the GFP signal intensity (Figure 6 C, left). In contrast, mutations in the TGCACA motifs resulted in only a slight reduction of the signal (Figure 6 C, left), indicating a higher contribution of the TGTCT motifs on the activation of *rsph9* than that of the TGCACA motifs. Nevertheless, the parasites in which both motifs were mutated showed weaker signal intensity than did the TGTCT motif mutant (Figure 6C, left). These two reporter assays strongly suggested that the TGCACA motif functions as an independent *cis*-regulatory element for the activation of downstream genes. Furthermore, the effect of disrupting the TGTCT motifs and both of the two motifs in the reporter assays was similar to the level of downregulation in *gsnf2*(−) and *pbarid*(−), respectively. This result is consistent with the implication that the differences between the phenotypes of *pbarid*(−) and *gsnf2*(−) could be due to the function of PbARID on the TGCACA motif.

### PbARID interacts with gSNF2 and other putative subunits of the SWI/SNF chromatin remodeling complex on the genome

**Figure 7.**
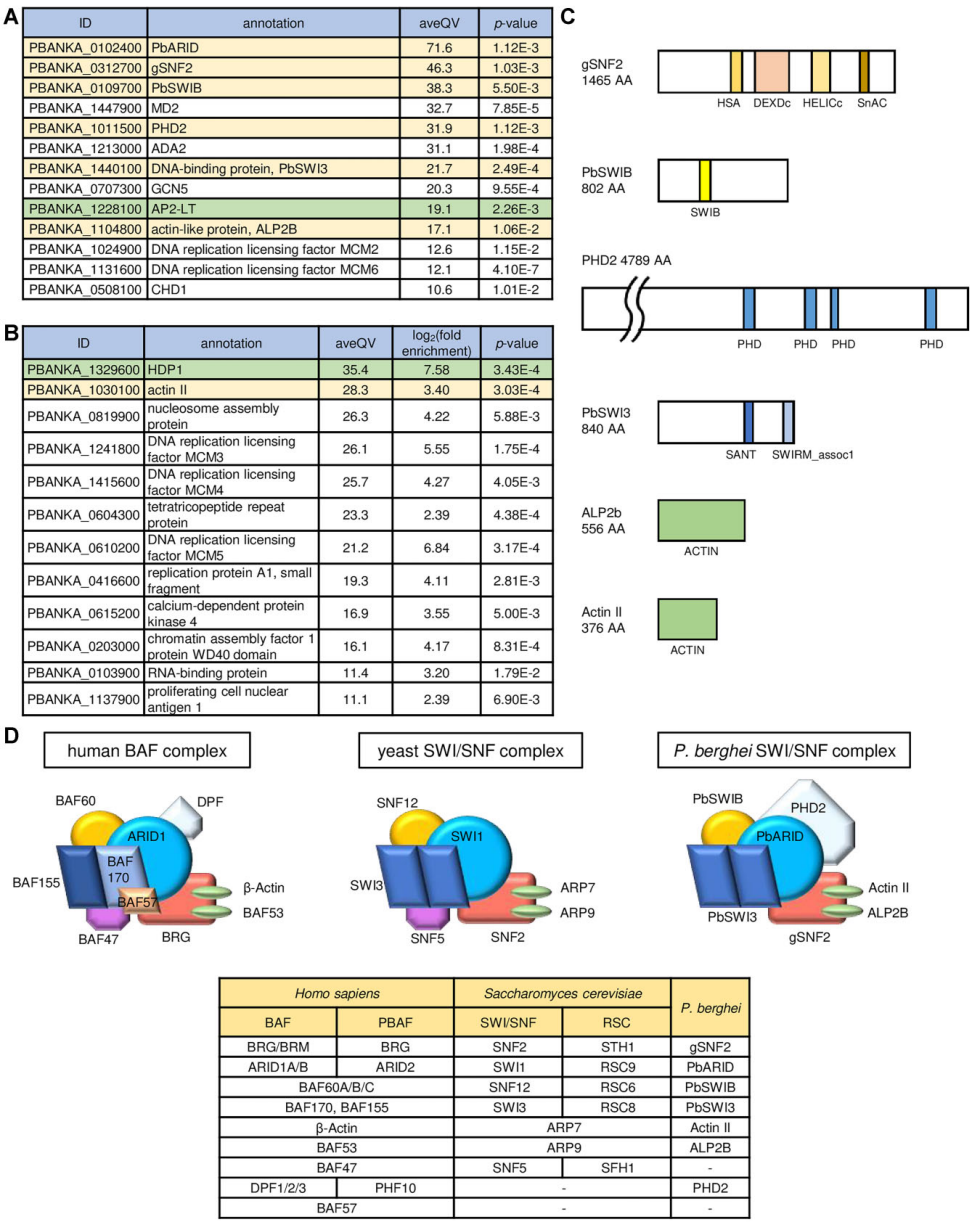
Rapid immunoprecipitation mass spectrometry of endogenous proteins (RIME) using PbARID::GFP. (**A**) A list of interaction partners of PbARID (PbARID-IPs) uniquely identified by the RIME of PbARID::GFP. Those with average quantitative value for PbARID::GFP (aveQV) > 10 are shown. Possible subunits of the SWI/SNF complex and transcription factors are indicated in yellow and green, respectively. (**B**) A list of PbARID-IPs five-fold enriched in the RIME of PbARID::GFP compared to that of wild-type. Those with aveQV >10 are shown. (**C**) Schematic illustrations of possible SWI/SNF subunits included in the PbARID-IPs. (HSA, helicase/SANT-associated; DEXDc, DEAD-like helicase domain; HELICc, helicase superfamily C-terminal domain; SWIB, SWI/SNF complex B/MDM2; PHD, plant homeodomain finger; SANT, Swi3, Ada2, N-Cor and TFIIIB; SWIRM_assoc1, Swi3p, Rsc8p and Moira-associated region 1; ACTIN, actin-related domain) (**D**) Schematic illustrations of the SWI/SNF complexes in humans, yeasts, and *Plasmodium berghei*. The table shows orthologs of each subunit across the three species.

To determine whether PbARID indeed forms a complex with gSNF2 on the genome, we performed rapid immunoprecipitation mass spectrometry (MS) of endogenous proteins (RIME), which is a method combining ChIP and MS, using PbARID::GFP. ChIP was performed as ChIP-seq analyses, and IPed proteins were on-beads digested with trypsin and subjected to liquid chromatography-tandem MS (LC-MS/MS) analysis. Four biologically independent experiments were performed, and the MS data were compared with the previous RIME data of WT gametocytes (35). Proteins that were unique or more than five-fold enriched, with *p*-value < 0.05, in PbARID::GFP were identified as possible interaction partners of PbARID (PbARID-IPs). The analysis revealed 366 and 445 proteins immunoprecipitated by the RIME using PbARID::GFP and WT, respectively. From these proteins, 58 PbARID-IPs (35 unique and 23 more than five-fold enriched proteins) were identified (Supplementary Table S5), including PbARID itself, which had the highest average quantitative value for PbARID::GFP (aveQV) (Figure 7A). Among the PbARID-IPs, gSNF2 had the second highest aveQV (Figure 7A). This result revealed that PbARID indeed interacts with gSNF2 on the genome, forming the SWI/SNF chromatin remodeling complex. Furthermore, PFG was detected in the PbARID-IPs (Supplementary Table S5), consistent with the ChIP-seq result showing that PFG recruits PbARID to the TGTAYRTACA motif.

Next, we searched for other possible subunits of the SWI/SNF complex in the PbARID-IPs. Here, we focused on PbARID-IPs with high aveQV (those with aveQV > 10 are shown in Figure 7A and B) and identified five subunit candidates besides PbARID and gSNF2 (Figure 7C): (i) PbSWIB (PBANKA_0109700), which had the third highest aveQV among the unique proteins, contains the SWIB (SWI/SNF complex B) domain (Figure 7A and C). The SWIB domain is conserved in orthologs of yeast SNF12, which are a subunit of the SWI/SNF complex (Figure 7D) (68). SNF12 orthologs form an initial core for assembling the SWI/SNF complex with dimerized SWI3 orthologs (69). (ii) PHD2 (PBANKA_1011500) has four plant homeodomain (PHD) fingers at its C-terminus (Figure 7A and C). The PHD finger recognizes post-translational histone modifications (70). The human and *Drosophila* SWI/SNF complexes contain a subunit that contains two tandem PHD fingers (58). Hence, PHD2 might be a subunit of the SWI/SNF complex in *Plasmodium*. In *P. falciparum*, PHD2 was reported to function in complex with a transcriptional coactivator, ADA2 (PBANKA_1213000) (71), and a histone acetyltransferase, GCN5 (PBANKA_0707300) (72), both of which were among the PbARID-IPs with high aveQVs (Figure 7A), implying association of PHD2 with the chromatin modifying complex (73). (iii) A putative DNA-binding protein (PBANKA_1440100) contains a SANT (Swi3, Ada2, N-Cor and TFIIIB) domain (Figure 7A and C). The SANT domain is conserved in SWI3 orthologs, a subunit of the SWI/SNF complex that constitutes the initial core complex. PBANKA_1440100 does not contain any detectable SWIRM (Swi3p, Rsc8p and Moira) domain, which is another conserved domain of SWI3 orthologs located at the N-terminal side of the SANT domain, but has SWIRM-associated region 1 (Pfam Domain: PF16495) (Figure 7C), one of the short regions that are conserved in SWI3 orthologs. Furthermore, orthologs of PBANKA_1440100 in apicomplexan parasites, which were identified using blastp search, contained the SWIRM domain at the N-terminal side of their SANT domain (Supplementary Figure S5A and S5B). When the amino acid sequence of PBANKA_1440100 was aligned with those of the blastp-detected proteins, PBANKA_1440100 had a region aligned with the SWIRM domain with some conserved amino acids (Supplementary Figure S5B). These indicated that PBANKA_1440100 is a SWI3 ortholog in *P. berghei*. Hence, we named PBANKA_1440100 as PbSWI3. 4) The actin-like protein, ALP2B (PBANKA_1104800), was identified with the 10th highest aveQV in the unique PbARID-IPs (Figure 7A and C). The SWI/SNF complex is known to interact with two actin-related proteins through the helicase/SANT-associated (HSA) domain of the ATPase subunit (74). As gSNF2 contains a HSA domain (40) (Figure 7B), the result indicated that ALP2B could be one of the actin-related proteins interacting with gSNF2. 5) Notably, actin II (PBANKA_1030100) was detected with the second highest aveQV in the five-fold enriched proteins (Figure 7B and C), suggesting that actin II could be another actin-related protein in the *Plasmodium* SWI/SNF complex. Collectively, the RIME detected seven putative subunits of the SWI/SNF complex, including PbARID and gSNF2 (Figure 7C). These includes six of seven subunits that are conserved among the SWI/SNF complexes of yeasts and humans, except for SNF5 orthologs (Figure 7D) (58,75). SNF5 is a component of the arm submodule in the SWI/SNF complex (76,77). Blastp search using the amino acid sequence of yeast SNF5 did not detect any homologous proteins in *Plasmodium*, suggesting that the *Plasmodium* parasites do not have SNF5 orthologs.

Next, we sought to explore transcription factors that may recruit PbARID to the TGTCT or TGCACA motifs. In the five-fold enriched proteins, homeodomain-like protein 1 (HDP1, PBANKA_1329600) was detected with the highest aveQV (Figure 7B). The homeodomain is a helix-turn-helix-related DNA binding domain (78). In *P. falciparum*, HDP1 functions during the early gametocyte development and is essential for both male and female gametocyte maturation (30). Another transcription factor candidate was an AP2-family transcription factor, AP2-LT (PBANKA_1228100) (Figure 7A). According to knockout screen studies, AP2-LT is essential for asexual blood-stage development in both *P. berghei* and *P. falciparum* (79,80). The expression of AP2-LT during gametocyte development was also indicated in *P. falciparum*, although its role has not been investigated (81).

In addition to these putative subunits of the SWI/SNF complex and transcription factors, we identified some developmental regulators in the PbARID-IP. MD2 (PBANKA_1447900) was detected with the fourth highest aveQV among the unique proteins (Figure 7A). MD2 was recently identified as an essential regulator for male gametocyte differentiation, as the single cell RNA-seq of *md2*-knockout parasite showed complete loss of male-differentiated cells (41). The role of MD2 in transcriptional regulation is unclear as no known functional domains could be detected in MD2. However, the fact that MD2 was co-IPed with PbARID suggests that MD2 is a nuclear factor. The PbARID-IPs also contained chromatin remodeling ATPases, namely chromodomain-helicase-DNA-binding protein 1 (CHD1, PBANKA_0508100) (Figure 7A and B) and, although with small aveQV (aveQV = 3.0), ISWI chromatin-remodeling complex ATPase (ISWI, PBANKA_1123500) (Supplementary Table S5). These ATPases might be involved in transcriptional regulation on the TGCACA motifs, which is independent of gSNF2.

### The transcription factor HDP1 binds to the TGCACA

**Figure 8.**
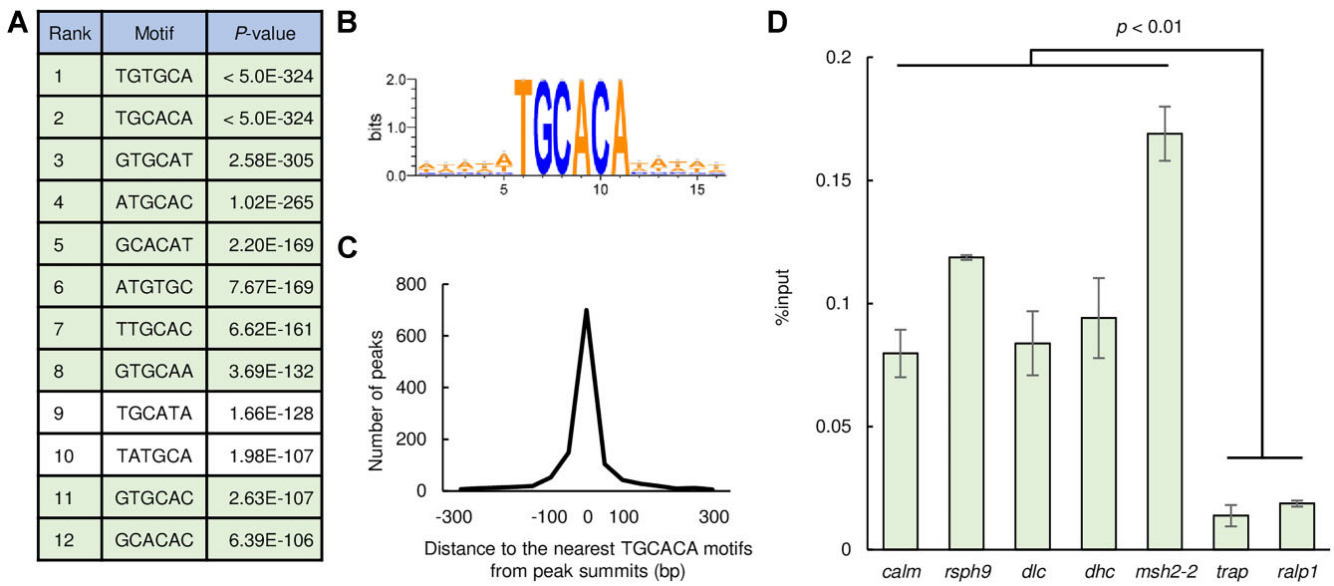
DNA-binding property of HDP1. (**A**) A list of 6-bp motifs enriched within 50 bp from peak summits identified in the DIP-seq of the HDP1 homeodomain. TGCACA/TGTGCA and corresponding motifs with one-base shifts are indicated by green. (**B**) The DNA motif most enriched in DIP-seq. The logo was depicted by WebLogo 3. (**C**) Distance between peak summits identified in DIP-seq and the nearest TGCACA motifs. (**D**) ChIP-qPCR analysis of HDP1 at the peaks identified through ChIP-seq, with PbARID::GFP^C_^*^gsnf2^*^(−)_^*^pfg^*^(−)^ upstream of dKO targets (*calm*, calmodulin; *rsph9*, radial spoke head protein 9 homolog; *dlc*, dynein light chain; *dhc*, dynein heavy chain; *msh2-2*, DNA mismatch repair protein MSH2) and other regions as negative controls (*trap*, thrombospondin-related anonymous protein; *ralp1*, rhoptry-associated leucine zipper-like protein 1). Error bars indicate the standard error of the mean %input values from three independent experiments.

Considering that HDP1 is essential for early gametocyte development in *P. falciparum* (30), we assumed that HDP1 was the most probable candidate for the sequence-specific transcription factor that recruits PbARID to the TGCACA motif. Based on this hypothesis, we performed DNA immunoprecipitation followed by high-throughput sequencing (DIP-seq) using recombinant *P. berghei* HDP1 homeodomain fused with glutathione S-transferase (GST-HDP1) to assess its binding motif. GST-HDP1 was mixed with the *P. berghei* genomic DNA fragmented via sonication (approximately 150−300 bp in size). Next, DNA fragments bound by GST-HDP1 were harvested using glutathione-sepharose resin and sequenced using NGS. From the sequence data, 1411 peaks were identified throughout the genome. Within 50 bp from the summit of these DIP-seq peaks, TGCACA and its reverse complement were enriched with *P*-values < 5.0 × 10^−324^ (the smallest positive real number on the R platform) using Fisher's exact test (Figure 8A and B). In addition, eight of the ten motifs most enriched following TGCACA/TGTGCA were those with one-base shifts (Figure 8A). Furthermore, in more than 80% of the DIP peaks (1177 peaks), the TGCACA motif was located within 300 bp from their summit, and most of them were located within 50 bp (Figure 8C). These results demonstrated that TGCACA was the binding motif of HDP1.

Next, we performed ChIP coupled with quantitative PCR (ChIP-qPCR) analysis of HDP1 to evaluate whether it binds to the TGCACA motifs upstream of the dKO targets. Parasites expressing GFP-fused HDP1 were developed using the CRISPR/Cas9 (HDP1::GFP, Supplementary Figure S6) and subjected to ChIP-qPCR at the gametocyte stage. Three biologically independent experiments were performed for the following analysis. The amount of immunoprecipitated DNA fragments relative to input DNA (%input value) was assessed for the peaks identified in the ChIP-seq analysis with PbARID::GFP^C_^*^gsnf2^*^(−)_^*^pfg^*^(−)^ upstream of several dKO targets [*calm*, *rsph9*, *dlc* (dynein light chain, PBANKA_0302200), *dhc* (dynein heavy chain, PBANKA_0416100), *msh2-2* (DNA mismatch repair protein MSH2, PBANKA_0804300)], which contain the TGCACA motif. The %input values for upstream of genes not expressed in gametocytes, *trap* (thrombospondin-related anonymous protein, PBANKA_1349800) and *ralp1* (rhoptry-associated leucine zipper-like protein 1, PBANKA_0619700), were also examined as the negative control. The analysis showed that DNA fragments for the upstream of all dKO targets assessed were enriched significantly, by more than four-fold with *p*-values < 0.01 as assessed via two-tailed Student's t-tests, compared to that of *trap* and *ralp1* (Figure 8D). Therefore, together with the RIME and DIP-seq analyses, the results suggested that HDP1 is the sequence-specific transcription factor that recruits PbARID to the TGCACA motif.

## Discussion

In this study, we demonstrated the essential role of PbARID in transcriptional regulation during the gametocyte development of *P. berghei*. The disruption of *pbarid* resulted in a complete loss of gametocytes with detectable male features and downregulation of most male-enriched genes, suggesting that PbARID is a major regulator of male development. The ChIP-seq analyses showed that PbARID is associated with gSNF2 at the TGTCT motif, which is a major *cis*-regulatory element for the activation of male genes. Along with the protein domain analysis of the ARID domain, this result suggested that PbARID functions as a subunit of the SWI/SNF chromatin remodeling complex for regulating male genes. However, the disruption of the SWI/SNF complex on the TGTCT motif does not completely explain the phenotype of *pbarid*(−) in male development; *i.e*. whereas *pbarid*(−) completely lost the ability to produce morphologically distinguishable male gametocytes, *gsnf2*(−) still produced immature male gametocytes. In addition, differential expression analyses between *pbarid*(−) and *gsnf2*(−) showed a significant difference between their gametocyte transcriptomes. Through the ChIP-seq using PbARID::GFP^C_^*^gsnf2^*^(−)_^*^pfg^*^(−)^, these differences were revealed to be derived from the function of PbARID on the TGCACA motif. Therefore, we considered that PbARID could be involved in two remodeling processes required for male gametocyte development, thereby associating with the two different chromatin remodeling complexes.

In female gametocytes, PbARID is recruited to the female *cis*-regulatory element, TGTAYRTACA, which is associated with a female-specific transcription factor, PFG. However, in contrast to the marked phenotype in male gametocyte development, *pbarid*(−) barely showed abnormality in female gametocytes, which only resulted in decreased efficiency of ookinete maturation. This phenotype of *pbarid*(−) is very different from that of the *pfg*-knockout parasite, in which the development of fertilized parasites is arrested at the zygote stage (33). Therefore, we considered that PbARID does not play an important role on the TGTAYRTACA motif, although recruited on it. In contrast, the target genes for the TGCACA motif (dKO targets), which are largely downregulated in *pbarid*(−), included a considerable number of female-enriched genes. Therefore, we speculate that the abnormality in female gametocytes of *pbarid*(−) is derived from the disruption of PbARID function on the TGCACA motif.

**Figure 9.**
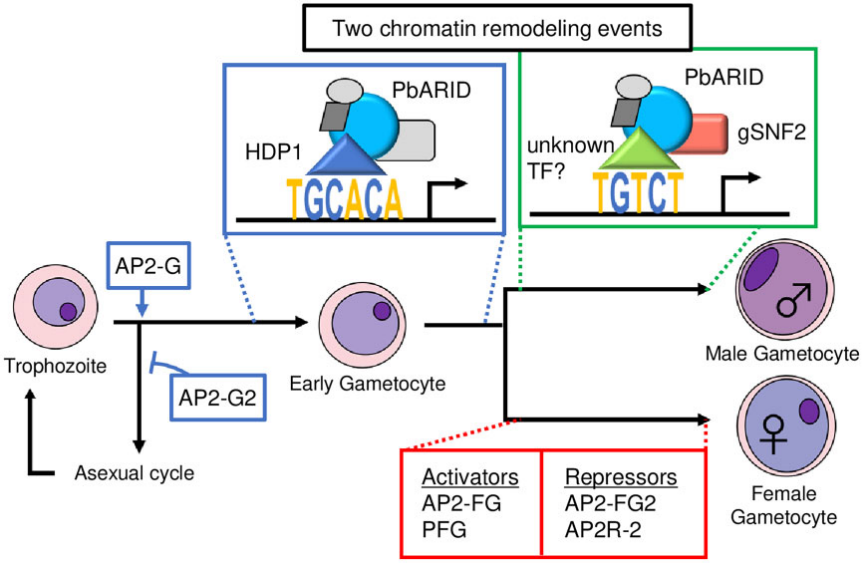
A schematic model for functions of PbARID during gametocyte development in *Plasmodium berghei*. First, AP2-G triggers gametocytogenesis, followed by AP2-G2 supporting gametocyte differentiation through global gene repression. Next, PbARID is recruited by HDP1 and activates early gametocyte genes on the TGCACA motif, cooperating in a chromatin remodeling complex that does not contain gSNF2. This remodeling complex is recruited by HDP1. After sex determination, male-gene transcription is further activated by the SWI/SNF chromatin remodeling complex containing gSNF2 and PbARID, associating with the TGTCT motif. For female development, transcriptional activators (AP2-FG and PFG) and transcriptional repressors (AP2-FG2 and AP2R-2) promote female gametocyte maturation.

The expression of PbARID begins before the manifestation of each sex characteristic, which is earlier than that of the sex-specific transcriptional regulators, gSNF2 and PFG. This indicates that PbARID functions before sex determination. Considering this and the roles of PbARID on the TGCACA motif discussed above, we propose a model that describes the functions of PbARID during gametocyte development (Figure 9). First, AP2-G triggers gametocytogenesis and activates gametocyte transcriptional regulator genes, including *pbarid*. Next, PbARID is recruited to the TGCACA motifs by HDP1 and activates early gametocyte genes, cooperating with a chromatin remodeler other than gSNF2. This step promotes early gametocyte development and preparation for male and female differentiation. In the male gametocyte lineage, male genes are activated via the SWI/SNF chromatin remodeling complex containing gSNF2 and PbARID, associating with the male *cis*-regulatory element, TGTCT. Some of the genes activated by the TGCACA motif are further transcribed to be expressed male-specifically under the control of the TGTCT motif. In *pbarid*(−), the functions of these two *cis*-regulatory elements are both impaired, thus resulting in the complete absence of male gametocytes. In the female gametocyte lineage, transcriptional activators, AP2-FG and PFG, activate female genes, recruiting PbARID. However, PbARID probably plays no important role in activating these female genes. A subset of the TGCACA targets is also activated by AP2-FG and PFG for female-specific expression. In *pbarid*(−), these activators complement the function of TGCACA in part, and hence, female gametocyte development in *pbarid*(−) is only mildly affected.

RIME analysis using PbARID::GFP detected possible subunits of the SWI/SNF complex. These included most of the subunits conserved in the SWI/SNF complexes of yeasts and animals, including the initial core subunits, SNF12 and SWI3 orthologs. This result suggested that formation of the *Plasmodium* SWI/SNF complexes proceeds similar to that of the SWI/SNF complexes in other species; *i.e*. dimerized PbSWI3 (SWI3 ortholog) forms an initial core with PbSWIB (SNF12 ortholog) (69,76) and interacts with the ATPase subunit through its SANT domain (77,82) (Figure 7C). Meanwhile, further investigation, such as confirming their genome-wide colocalization by ChIP-seq, would be required to determine the accurate complex associated with PbARID. According to PlasmoGEM knockout screen, PbSWIB and PbSWI3 are essential for the asexual blood stage development (83). This implies that chromatin remodeling could also be important for the asexual blood-stage development. Moreover, in *P. falciparum*, PfSWIB was reported to play a role in transcriptional regulation of *var* genes, suggesting the role of chromatin remodeling in subtelomeric multigene regulation (84). We expect that investigation of these subunits would help identify other chromatin remodeling factors important for asexual blood stages and elucidate the mechanisms regulating virulence genes.

Apart from the chromatin remodeling factors, the transcription factor HDP1 was identified in the PbARID-IPs. DIP-seq and ChIP-qPCR analyses confirmed HDP1 as the transcription factor that recruits PbARID to the TGCACA motif. In *P. falciparum*, HDP1 binds to GTGCAC, a motif with a one-base shift in the TGCACA, and plays an essential role in both male and female gametocyte maturation (30). Thus, HDP1-mediated transcriptional regulation during early gametocyte development could be conserved in *Plasmodium*. Meanwhile, the role of TGCACA in *P. berghei* gametocyte development has not been fully elucidated because the disruption of *pbarid* affected transcriptional regulation at both the TGCACA and TGTCT motifs. Therefore, further investigating the function of HDP1 in *P. berghei* gametocyte development is important. Moreover, a chromatin remodeling ATPase that functions with HDP1 at TGCACA remains to be unidentified. The candidate for this HDP1-associated ATPase is two chromatin remodeling ATPases, CHD1 and ISWI, detected in the PbARID-IPs. CHD1 was identified as a unique protein with a high aveQV, implying that it is a strong candidate for a PbARID-associated remodeler on the TGCACA motif. However, unlike other ATP-dependent remodelers, CHD subfamily remodelers do not form a multi-subunit complex, but rather associate with chromatin-modifying factors, such as GCN5 and FACT (85). In contrast, the ISWI complex in *Arabidopsis* contains ARID5, as an essential subunit (86,87), suggesting that *Plasmodium* ISWI might be the remodeler that cooperates with PbARID on the TGCACA motif. Assessing the nuclear complex associated with HDP1 using RIME would reveal which of these ATPases is involved in the transcriptional regulation at the TGCACA.

The RIME also detected other nuclear factors. Two chromatin modifiers, GCN5 and ADA2, function as a histone acetyltransferase (HAT) complex that acetylates H3K9 to activate gene expression in *P. falciparum* (71,72,88). They also interact with PHD2 and AP2-LT and are involved in transcriptional regulation during asexual blood-stage development (89). In yeast, HATs are associated with SWI/SNF complexes and facilitate their recruitment or chromatin remodeling for transcriptional activation (90,91). Therefore, the SWI/SNF complex and HAT complex could cooperate to activate downstream genes during gametocyte development in *Plasmodium*. Furthermore, the PbARID-IPs included the male differentiation regulator MD2. Previously, single-cell RNA-seq had revealed that MD2 is essential for male differentiation but not involved in female development (41). While MD2 expression has not been evaluated *in vivo*, our RIME suggested that MD2 is a nuclear factor. Thus, MD2 may be associated with transcriptional activation at the major male *cis*-regulatory element, TGTCT.

To conclude, we revealed the functions of PbARID as a subunit of chromatin remodeling complexes. Moreover, our results showed that PbARID functions with gSNF2 on the TGTCT motif and also with another, yet unknown, remodeler on the TGCACA motif. Our results further showed that chromatin remodeling events could be important for both male and female development of *P. berghei*. In a recent study, disruption of the *pbarid* ortholog in *P. falciparum* was also shown to affect both male and female development (44). Therefore, the PbARID-mediated chromatin remodeling processes for early and male gametocyte development could be conserved in *Plasmodium* species. To deeply understand the mechanism of transcriptional regulation during the *Plasmodium* gametocyte development, further exploration of other transcriptional regulators, especially those identified by our RIME, is required.

## Supplementary Material

gkae207_Supplemental_Files

## Data Availability

All data produced in this study are fully available without restriction. All FASTQ files for ChIP-sequencing, RNA-sequencing and DIP-sequencing experiments are available from the Gene Expression Omnibus database (accession numbers GSE235412 and GSE256093). All MS/MS data for RIME are available from the ProteomeXchange Consortium (dataset PXD047471).
