# Supplementary material for: PbARID-associated chromatin remodeling events are essential for gametocyte development in Plasmodium
Source: Nucleic Acids Res. 2024 Mar 30;52(10):5624–42. doi: 10.1093/nar/gkae207 (PMC11162789; doi:10.1093/nar/gkae207)
Supplement: gkae207_Supplemental_Files [file gkae207_supplemental_files.zip › Supplementary Data_2.pdf]

Supplementary Data

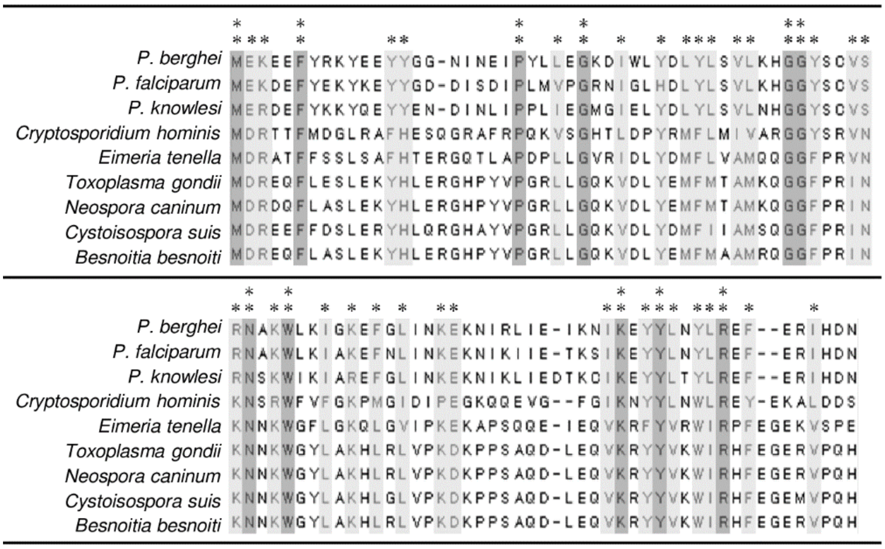

Fig S1. Alignment of amino acid sequences for PbARID and its putative orthologs in apicomplexan parasites by the ClustalW program in Mega X

(*Plasmodium berghei*, PbARID; *Plasmodium falciparum*, PF3D7_0603600; *Plasmodium knowlesi*, PKNH_1147100; *Cryptosporidium hominis*, XP_665701; *Eimeria tenella*, XP_013235762; *Toxoplasma gondii*, XP_018634911; *Neospora caninum*, XP_003885918; *Cystoisospora suis*, PHJ24466; and *Besnoitia besnoiti*, XP_029222326)

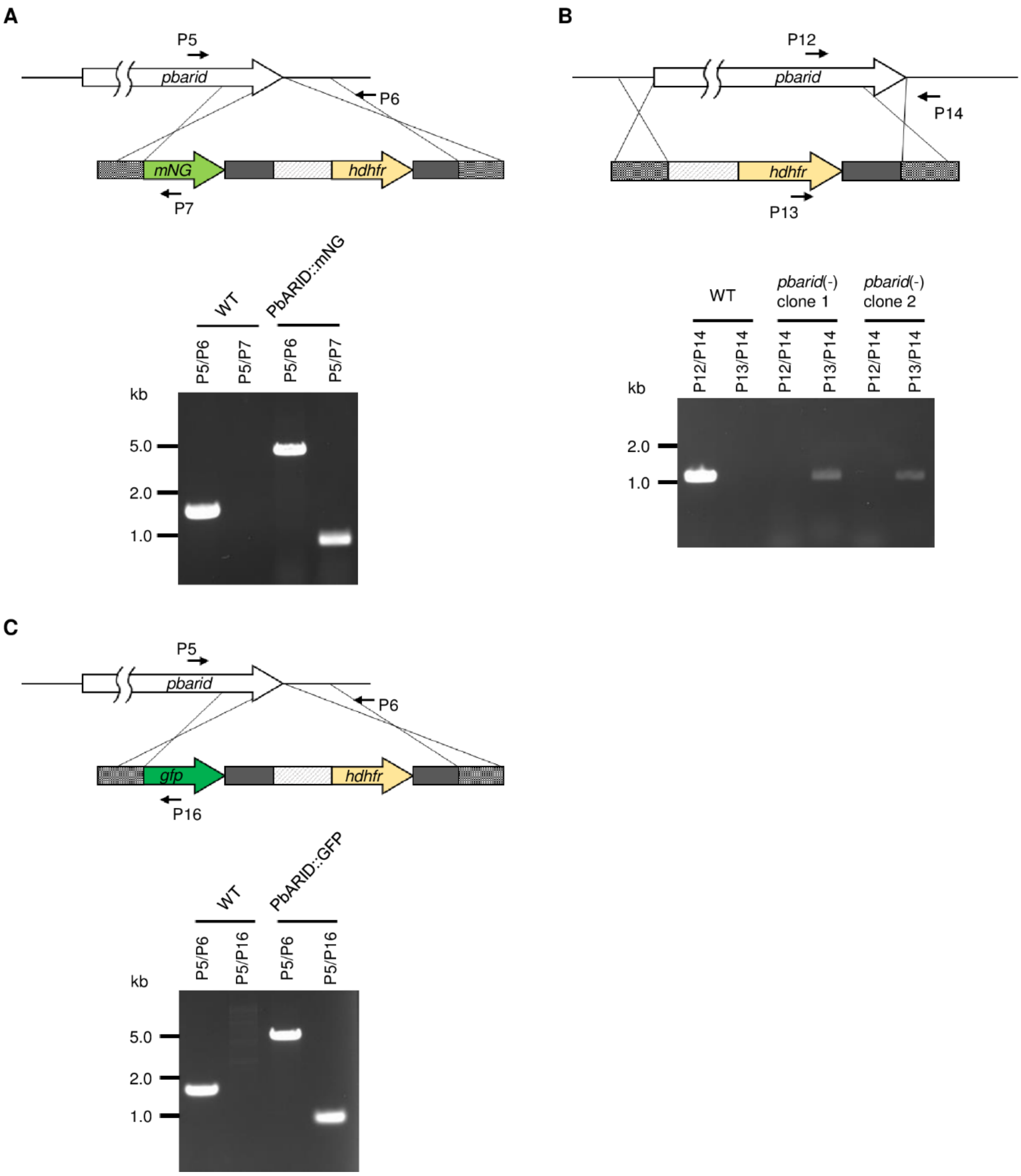

Fig S2. Genotyping of transgenic parasites used for expression and phenotype analyses of *pbarid*

(A) PbARID::mNG. (B) *pbarid*(−). (C) PbARID::GFP.

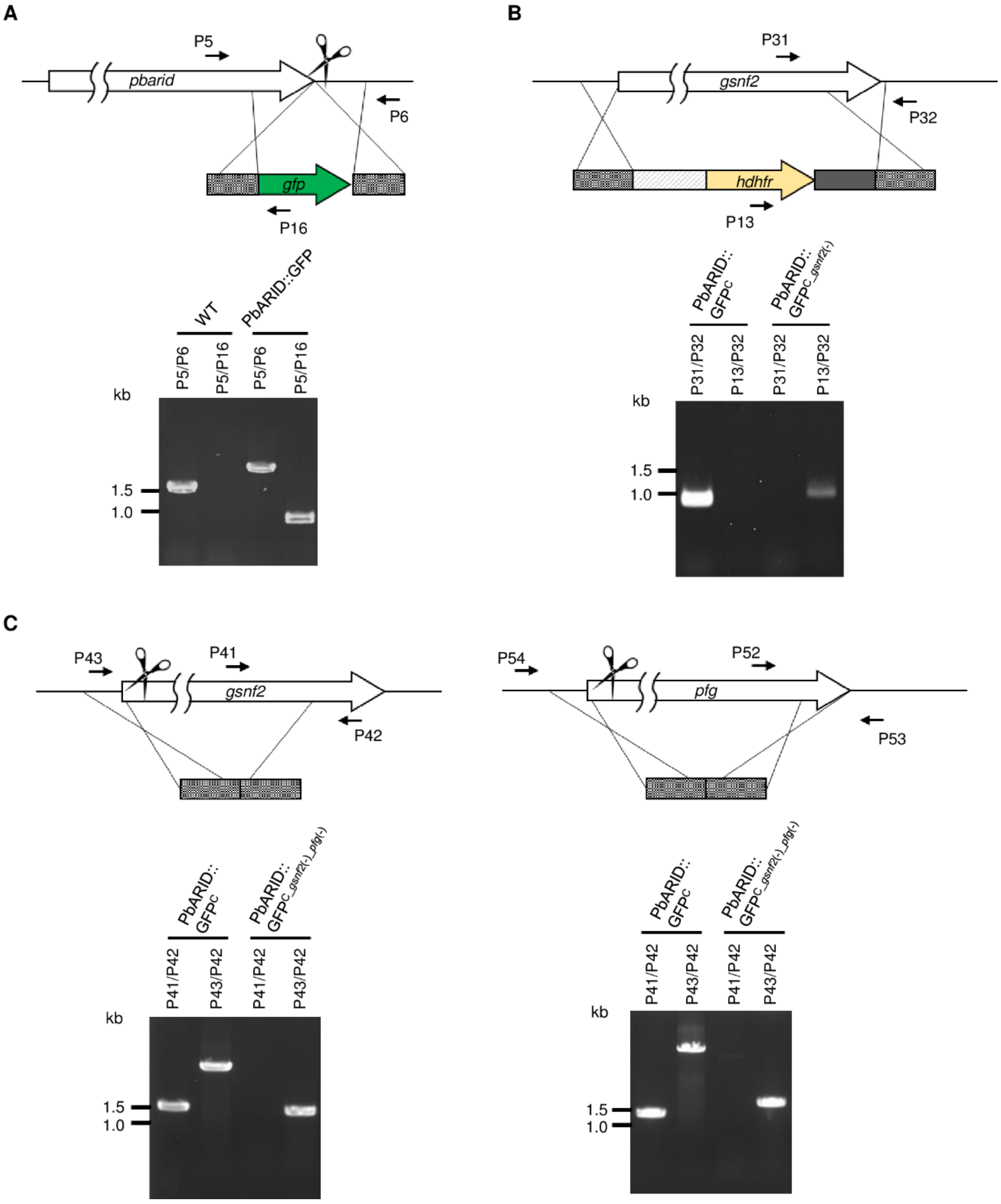

Fig S3. Genotyping of transgenic parasites used for ChIP-seq experiments

(A) PbARID::GFP^{C}. (B) PbARID::GFP$^{C_gsnf2(-)}$. (C) PbARID::GFP$^{C_gsnf2(-)_pfg(-)}$.

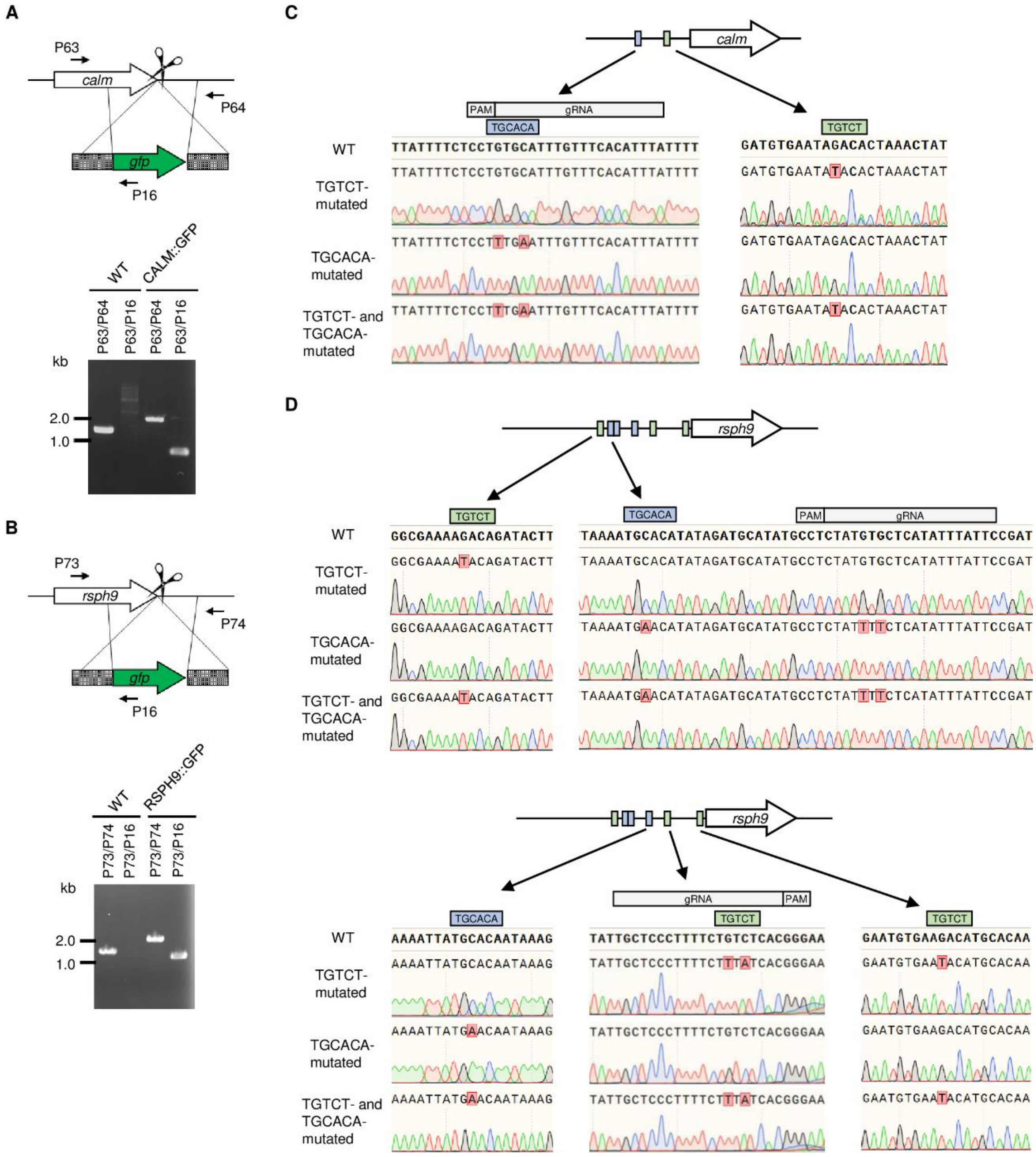

Fig S4. Genotyping of transgenic parasites used for reporter assays

(A) CALM::GFP. (B) RSPH9::GFP. (C) CALM::GFP^{motif_mutated}. (D) RSPH9::GFP^{motif_mutated}.

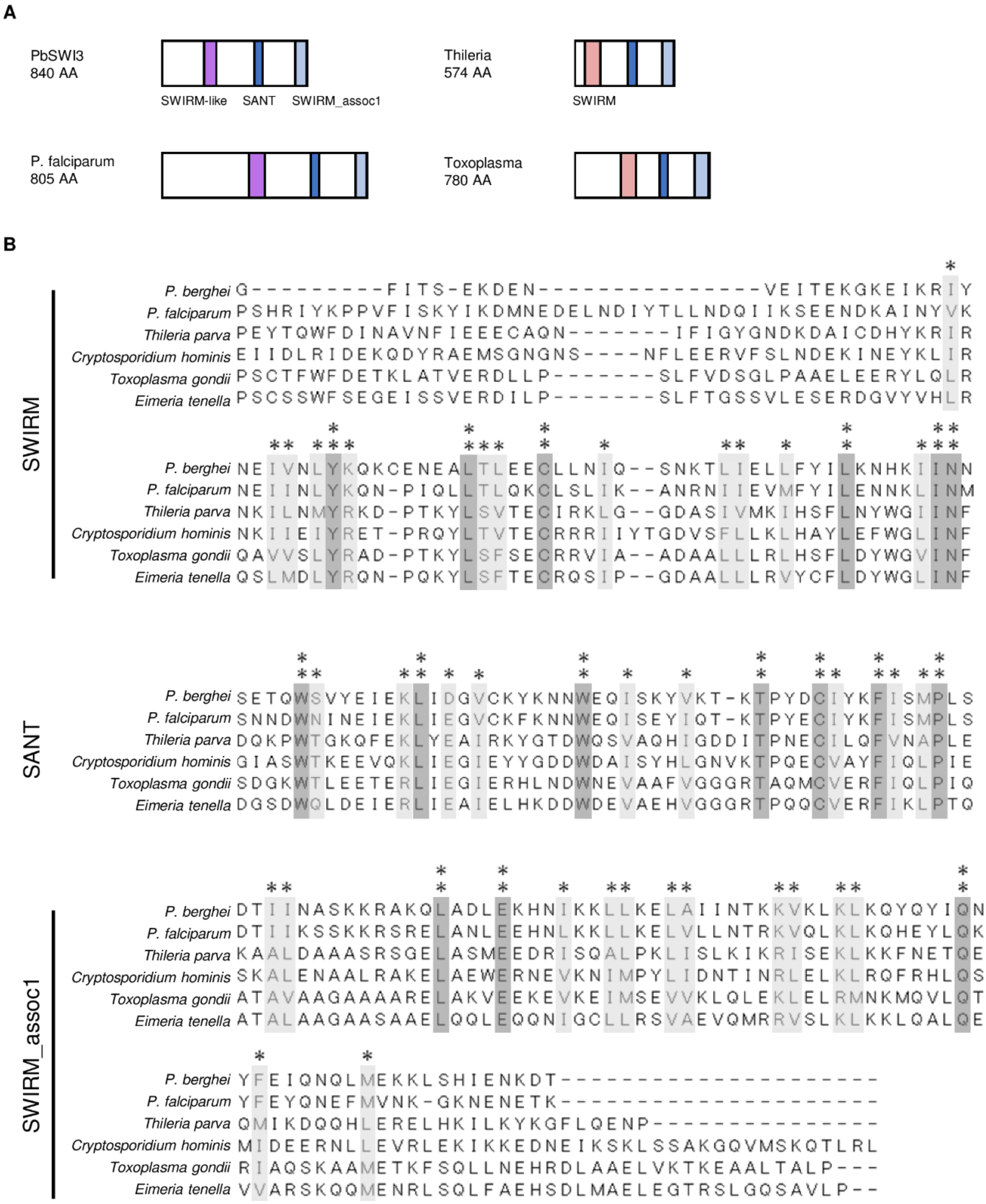

Fig S5. PbSWI3 orthologs in Apicomplexa

(A) Schematic illustration of PbSWI3 and its putative orthologs in Apicomplexa. (SWIRM, Swi3p, Rsc8p, and Moira: SANT, Swi3, Ada2, N-Cor, and TFIIIB: SWIRM_assoc1, SWIRM-associated region 1). (B) Alignment of amino acid sequences for PbSWI3 and its putative orthologs in Apicomplexa using the ClustalW program in Mega X. Regions for SWIRM, SANT, and SWIRM_assoc1 are shown. (*Plasmodium*

berghei, PbSWI3; *P. falciparum*, PF3D7_1225200; *Theileria parva*, XP_762838; *Cryptosporidium hominis*, OLQ19103; *Toxoplasma gondii*, XP_002369308; *Eimeria tenella*, XP_013232460)

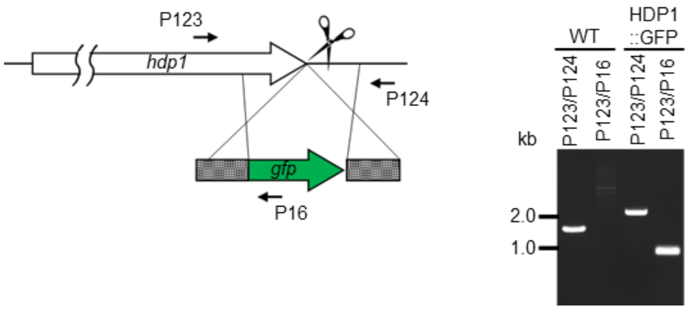

Fig S6. Genotyping of HDP1::GFP

Table S1. Differential expression analysis between *pbarid*(−) and wild-type

Table S2. ChIP-seq analysis of PbARID

(A) Experiment 1. (B) Experiment 2. (C) Target genes.

Table S3. Differential expression analysis between *pbarid*(−) and *gsnf2*(−)

Table S4. ChIP-seq analysis of PbARID using PbARID::GFP$^{C_gsnf2(-)_pfg(-)}$

(A) Experiment 1. (B) Experiment 2. (C) dKO targets.

Table S5. RIME analysis using PbARID::GFP and WT

Table S6. List of primers used in this study
